# Morphological and Rheological Properties of PLA, PBAT, and PLA/PBAT Blend Nanocomposites Containing CNCs

**DOI:** 10.3390/nano11040857

**Published:** 2021-03-27

**Authors:** Mojtaba Mohammadi, Marie-Claude Heuzey, Pierre J. Carreau, Aurélie Taguet

**Affiliations:** 1 Center for High Performance Polymer and Composite Systems (CREPEC), Department of Chemical Engineering, École Polytechnique de Montréal, Montreal, QC H3T 1J4, Canada; mojtaba.mohammadi@polymtl.ca (M.M.); marie-claude.heuzey@polymtl.ca (M.-C.H.); 2 Polymers Composites and Hybrids (PCH), IMT Mines Ales, 30319 Ales, France; aurelie.taguet@mines-ales.fr

**Keywords:** cellulose nanocrystals, PLA, PBAT, CNC localization, blends, nanocomposites, rheology, morphology

## Abstract

Morphological and rheological properties of poly(lactic acid), PLA (semicrystalline and amorphous), and poly(butylene adipate-co-terephthalate), PBAT, and their blends (75 wt%/25 wt%; PLA/PBAT) were investigated in the presence of cellulose nanocrystals (CNCs) prepared from solution casting followed by melt mixing. For the solution casting step, the CNCs were either incorporated into the matrix, the dispersed phase, or both. The dispersion and distribution of the CNCs in the neat polymers and localization in their blends were analyzed via scanning electron microscopy (SEM) and atomic force microscopy (AFM). The highly dispersed CNCs in the solution cast nanocomposites were agglomerated after melt mixing. In the blends with 1 wt% CNCs, the nanoparticles were mostly localized on the surface of the PBAT droplets irrespective of their initial localization. The rheological behavior of the single polymer matrix nanocomposites and their blends was determined in dynamic and transient shear flow in the molten state. Upon melt mixing the complex viscosity and storage modulus of the solution cast nanocomposites decreased markedly due to re-agglomeration of the CNCs. Under shearing at 0.1 s^−1^, a significant droplet coalescence was observed in the neat blends, but was prevented by the presence of the CNCs at the interface in the blend nanocomposites.

## 1. Introduction

Over recent years, poly(lactic acid) (PLA) has received remarkable attention mainly because it is a bio-based, biodegradable under specific conditions, biocompatible, and non-toxic polymer [1]. However, PLA suffers from serious drawbacks such as low melt strength, low and slow crystallization rate, poor processability, low toughness, low service temperature, and high brittleness [2]. Polymer blending is one of the most commonly used and practical approaches to improve the properties of PLA [3,4]. One of the most promising polymers to blend with PLA is poly(butylene adipate-co-terephthalate) (PBAT) with high flexibility and ductility features [5]. Jalali Dil et al. [6] investigated the morphology, miscibility and co-continuity development of a PLA/PBAT blend. They showed that the co-continuity region of the PLA/PBAT blend starts at a PBAT volume fraction between 30 and 40% [6]. Different studies revealed a low interfacial tension of around 1 mN/m for the PLA/PBAT system [7].

The final performance of polymer blends can be increased by introducing nanoparticles as reinforcements [8,9]. The localization of nano-inclusions at the interface, in the matrix, or dispersed phase can have a significant effect on the blend properties [8,10]. These localizations are affected by thermodynamics [11] and processing parameters such as the sequence of mixing [12,13], the viscosity of polymer components [13,14] as well as the quality of the particle dispersion and nature of the particles [15].

Different localizations have been investigated through the incorporation of different nanoparticles in PLA/PBAT blends such as nano-silica [13,16], carbon nanotube [12,17,18], graphene [19,20], and nano-clay [19,21,22,23,24]. Jalali Dil et al. [16] investigated the droplet/matrix and co-continuous morphology of PLA/PBAT (70/30 and 50/50, respectively) in the presence of nano-silica. They reported that adding 1 wt% nano-silica decreased the droplet size from 1.7 to 1 µm and by increasing the amount of nano-silica the droplet-like morphology changed to a co-continuous state. Nofar et al. [23] investigated properties of 75/25 (wt%) PLA/PBAT blends containing an organo-modified nano-clay (Cloisite 30B). Similarly to thermodynamics predictions, the organoclay was located at the interface of the two phases, was found to act as a barrier against the coalescence of droplets and stabilized the blend morphology under shear flow. Salehiyan et al. [25] also investigated the effects of selective localization of 1 wt% of carbon nanotubes, nano-silica, nano-clays, and graphene oxides on the morphology development and rheological properties of melt-processed PLA/PBAT blend nanocomposites.

One of the most promising nanoparticles is cellulose nanocrystal (CNCs), which is based on one of the most abundant resources in the environment and has the advantages of being non-toxic, biocompatible, and biodegradable. CNC has been used to increase the properties of various polymers, in particular PLA and PBAT. In our research group, solution casting methods were used to improve the morphology, rheological and mechanical properties of PLA/CNC and PBAT/CNC nanocomposites [26,27,28,29,30]. Bagheriasl et al. [26] used dimethylformamide (DMF) to prepare PLA/CNC nanocomposite and for the first time obtained a high degree of dispersion of pristine CNCs in PLA and reached a rheological percolation threshold at 0.66 wt% CNCs. Mohammadi et al. [30], based on a thermodynamics analysis, also identified that dimethyl sulfoxide (DMSO) and tetrahydrofuran (THF) were the best solvents for the dispersion of the CNCs and dissolution of semicrystalline PLA (scPLA) and amorphous PLA (aPLA)) as well as PBAT. They obtained the lowest rheological percolation threshold of 0.3 wt% CNCs in scPLA and PBAT and 1 wt% in aPLA [30]. They also showed that the complex viscosity dramatically decreased by one to two orders of magnitude for PLA due to the presence of residual solvent, but residual solvent did not affect PBAT, probably due to crystallization of the latter at the drying temperature (70 °C) [30].

There are many pieces of research on PLA blends containing CNCs. It can be categorized as PLA/poly-hydroxybutyrate (PHB)/freeze-dried-CNC [31,32,33], PLA/ polybutylene succinate (PBS)/CNC [34,35], PLA/poly-vinyl alcohol (PVAc)/CNC [36], and PLA/natural rubber (NR)/CNC [37,38]. However, none of these reports a very good dispersion of CNCs as shown by transmission electron microscopy (TEM) or atomic force microscopy (AFM) images. In a recent study, Sarul et al. [39] investigated the preparation of PLA/PBAT/CNC blend nanocomposites through solution casting followed by melt mixing via a twin-screw extruder. However, the authors did not report on the CNC dispersion in the neat polymers. Their analysis of the effect of the localization of CNCs was based on expectations from thermodynamics considerations and they did not present a microscopic analysis to localize the CNCs and confirm their thermodynamics analysis. They did not present a strong explanation on the rheological analysis section (with no information about the rheological properties of the single polymer matrices before and after melt mixing). Heshmati et al. [40] reported a very good dispersion of spray-dried CNCs in PLA/PA11 blends, prepared through a combination of solvent casting and melting methods. They also prepared a masterbatch of both PLA/CNC and PA11/CNC and diluted them via melt mixing. They showed that irrespective of the preparing method the spray dried CNCs preferred to remain in the PA11 phase, which was the thermodynamically favorable phase. Heshmati et al. [41] also showed that using poly(ethylene oxide) (PEO) as a polymer carrier for CNCs in the blend of PLA/PA11 resulted in the localization of the CNCs in PLA, which was not the thermodynamically favorable phase for the CNCs.

The goal of this work is to investigate the effect of melt mixing on rheology and morphological properties of highly dispersed CNCs of solution cast PLA-based nanocomposites. Droplet coalescence during processing is avoided by controlling the localization of CNCs in PLA/PBAT blends. The blend composition was chosen as 75 wt% PLA and 25 wt% PBAT in order to have an emulsion-type (droplets) morphology while the concentration of PBAT is large enough to significantly affect the rheological properties of the blends.

## 2. Materials and Methods

### 2.1. Materials

Ingeo 4060D and 3251D were used as the amorphous PLA (aPLA) and the semicrystalline PLA (scPLA), respectively. They were purchased from NatureWorks LLC (Minnetonka, MN, USA). The PBAT (Ecoflex^®^ FBX 7011) was purchased from BASF (Montreal, QC, Canada). The aPLA has a weight average molecular weight of 190 kg/mol and a D-lactide content of 12 mol%, and scPLA has a weight average molecular weight of 55 kg/mol and a D-lactide content of 1.4 mol%. The PBAT has a weight average molecular weight of 24.4 kg/mol, a density of 1.23 g/cm^3^, and a melt flow index (MFI) of 2 g/10 min. Freeze-dried CNCs were kindly provided by FP Innovations (Pointe-Claire, QC, Canada) with width, length, and aspect ratio of 16 ± 3, 90 ± 17 nm, and 6 ± 2, respectively [26]. Information on CNC preparation can be found elsewhere [42]. These CNCs were neutralized using sodium hydroxide (NaOH) before freeze-drying. N,N-dimethylformamide (DMF), anhydrous 99.8 %, was purchased from Sigma-Aldrich Canada Co. (Oakville, ON, Canada).

### 2.2. Single Polymer Matrix and Blend Nanocomposites Preparation

#### 2.2.1. Single Polymer Matrix Nanocomposites Preparation

DMF was used to disperse and dissolve the CNCs and the neat polymers using a water bath sonicator and magnetic stirrer, respectively. After complete dispersion and dissolution of CNCs and neat polymers, they were further mixed using a magnetic stirrer. Then, the mixtures were poured into a petri dish and dried in an oven in two steps under air circulation and vacuum (the details and step by step preparation method is presented in the Supplementary Materials (SM)). The weight percentage of CNC within the nanocomposites was 0 (i.e., neat polymers for a comparison purposes), 1, and 3. The CNC content was reported based on weight percentage basis. In this regard, a PLA/3CNC denotes a nanocomposite made of the amorphous (high molecular weight) PLA containing 3 wt% CNCs, calculated as a percentage of total weight of the nanocomposites. The effect of melt mixing using a DDRV501 Brabender (C. W. Brabender Instruments Inc., South Hackensack, NJ, USA) was also investigated on previously dried single polymer matrix nanocomposites, operating at 180 °C, 100 rpm for 7 min under a nitrogen atmosphere. The term “+IMM” is used in the nomenclature to identify the effect of melt mixing on the samples from solution casting.

#### 2.2.2. Blend Nanocomposites Preparation

Blend nanocomposites containing 75 wt% PLA and 25 wt % PBAT and, overall, 1 wt % CNCs were prepared from the nanocomposites as described above using the internal mixer (at 180 °C, 100 rpm for 7 min under a nitrogen atmosphere) and the detailed formulations are provided in Table 1. The schematic preparation method is provided in the SM (Figure S1). In the first two mixing strategies, granules of the neat complementary polymer (dried overnight at 55 °C) were added to the polymer nanocomposites in the internal mixer. In the third strategy both PLA and PBAT nanocomposites containing 1 wt% prepared from solution casting were melt mixed in the internal mixer. For example, (PLA-1CNC)/PBAT (mixing strategy 1) represents the blend nanocomposites containing 1 wt% CNCs based on the whole blend for which the CNCs were initially localized in the matrix (PLA). Similarly, PLA/(PBAT-1CNC) and PLA/PBAT/1CNC refer to the blend nanocomposites when the CNCs were initially localized in the dispersed (PBAT) phase or both phases, respectively. Three different neat blends were prepared for comparison purposes: neat PLA/PBAT blends from the granules (melt mixing), neat blends from solution casting, and neat blends from solution casting followed by the internal melt mixing.

A hydraulic press was used to prepare the rheological disk shape with 1.2 mm thickness and 25 mm in diameter. The compression molding process continued for 10 min at 180 °C under a nitrogen atmosphere including 4 min of heating and 6 min of progressive increasing pressure force from 1 to 3 tons. The rheological disk shapes were used for microscopy analysis.

### 2.3. Characterization

#### 2.3.1. Scanning Electron Microscope (SEM)

In order to determine the morphology and the localization of cellulose nanocrystals, the blends and blend nanocomposites were fractured in liquid nitrogen. A chromium-coated layer of 15 nm thickness was then applied to the samples. The morphology was observed under SEM (JSM 7600F, JEOL, Akishima, Tokyo 196-8558, Japan) at a voltage of 5 kV. The blend nanocomposites were also observed (after cryofracture of a thickness of about 20 nm) using an environmental scanning electron microscope Quanta 200 FEG from FEI company, SEM operating at 3 kV.

The volume-average radius (*R_v_*) of the dispersed phase domains was defined as follows:

(1) $$R_{v}=\frac{\sum_{i}n_{i}R_{i}^{4}}{\sum_{i}n_{i}R_{i}^{3}}$$

where *n_i_* is the number of dispersed domains with radii *R_i_* counted from SEM images [43], for at least 250–350 PBAT droplets, using the ImageJ software (version 1.52a Wayne Rasband, National Institutes of Health, Bethesda, MD, USA). As the samples were fractured in liquid nitrogen, no correction was applied to account for the fact that the observation plane might not cut the particles through their equator. In the samples with dispersed elongated droplets, an equivalent radius (*R_eq_*) of an oval was used and calculated as follows [44]:

(2) $$R_{eq}=\frac{3.1A^{0.625}}{P^{0.25}}$$

(3) $$\mathrm{with}\;\;\;\;\;\;\;A=\frac{\pi ab}{4}$$

(4) $$\mathrm{and}\;P\sim 2\pi \sqrt{\left(\frac{1}{2}\left({\left(\frac{a}{2}\right)}^{2}+{\left(\frac{b}{2}\right)}^{2}\right)\right)}$$

where *A* and *P* are the cross-section area and perimeter of the ovals, respectively, and *a* and *b* are major and minor dimensions of the flat ovals, respectively [44]. Using Equations (1)–(4), the equivalent volume-average radius (*R_v-eq_*) was calculated for the samples with elongated droplets.

#### 2.3.2. Atomic Force Microscopy (AFM)

Samples were cut and micro-tomed using an Ultracut FC microtome (Leica, Jung RM 2165, Concord, Ontario, Canada) equipped with a liquid nitrogen cryo-chamber and a glass knife. AFM images were acquired in the air at room temperature without any additional preparation using tapping mode on a Dimension ICON AFM (Bruker/Santa Barbara, CA, USA). Intermittent contact imaging (i.e., “tapping mode”) was performed at a scan rate of 0.8 Hz using etched silicon cantilevers (ACTA from AppNano, Mountain View, CA, USA) with a resonance frequency of around 300 kHz, a spring constant of ≈ 42 N/m, and a tip radius of <10 nm. All images were acquired with a medium tip oscillation damping (20–30%).

#### 2.3.3. Rheometry

The rheological properties of the neat aPLA, scPLA, PBAT, and their respective neat blends and nanocomposites were measured using a stress/strain-controlled MCR 302 rheometer (Anton Paar, Graz, Austria). A parallel plate flow geometry was used with a gap of 1 mm and a diameter of 25 mm. All rheological experiments were conducted at 180 °C under a nitrogen atmosphere to avoid oxidation of the samples. Strain sweep tests were conducted at a frequency of 1 rad/s to find the linear viscoelastic region (LVE) and all small amplitude oscillatory shear (SAOS) tests were conducted at a strain amplitude of 0.001. Time-sweep experiments at a frequency of 1 rad/s were carried out for 40 min to verify the thermal stability of the samples within the time necessary to conduct the frequency sweep experiments, all done from 628 rad/s to 0.05 rad/s. The structural recovery of the nanocomposites was investigated, following consecutive stress-growth experiments at a shear rate of 5 s^−1^, via time sweep experiments at 1 rad/s for 1800 s, and frequency sweep experiments. For the blend nanocomposites, stress-growth experiments were carried out at a shear rate of 0.1 s^−1^, selected to investigate coalescence of droplets in the blends (at that low shear rate, no droplet break-up is expected, as the capillary number, Ca, should be smaller than 1 [45]). Also, coalescence in PLA/PBAT/CNC blend nanocomposites was analyzed through SAOS time sweep experiments at a frequency of 1 rad/s for 1 h. Almost all rheological measurements were repeated up to three times to verify reproducibility.

## 3. Results and Discussion

### 3.1. Neat PLA and BPAT Nanocomposites

#### 3.1.1. Dispersion of CNCs in PLA and PBAT Matrices

**Figure 1:**
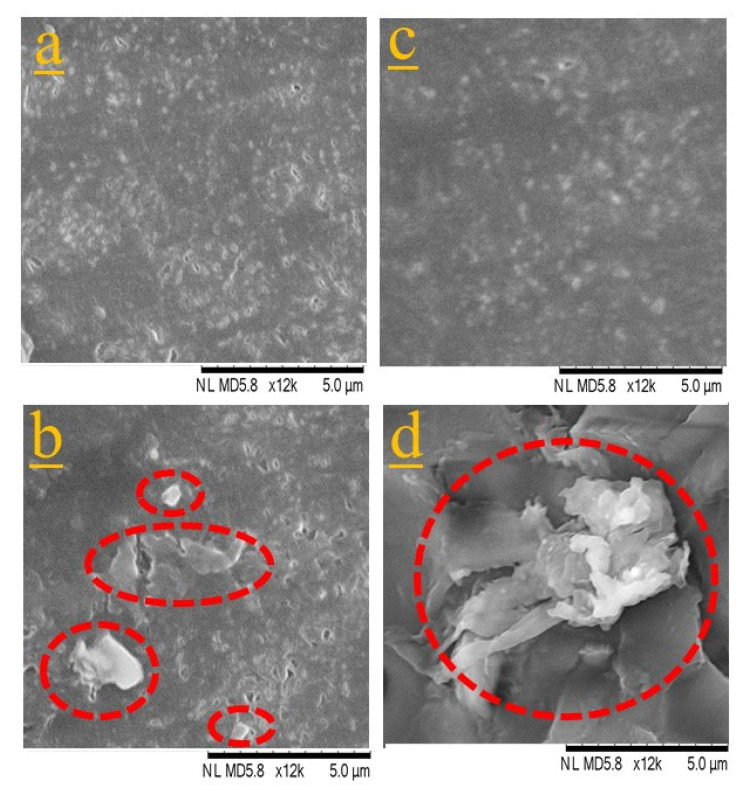
Scanning electron microscopy (SEM) images showing the dispersion and distribution of CNCs in (**a**,**c**) scPLA/1CNC and aPLA/1CNC nanocomposites, respectively, prepared from solution casting, in (**b**,**d**) after melt mixing for scPLA/1CNC and aPLA/1CNC nanocomposites, respectively.

Figure 1 shows SEM micrographs of scPLA/1CNC and aPLA/1CNC nanocomposites from solution casting before melt mixing (Figure 1a,c) and after melt mixing (Figure 1b,d). Solution casting leads to a good dispersion and distribution of the CNCs in both PLAs (Figure 1a,c), more likely as small bundles than individual CNC nanorods. Comparing Figure 1a–d, it is obvious that melt mixing leads to the agglomeration of CNCs (circles in Figure 1b,d). The agglomeration of CNCs is more important in the high molecular weight PLA (aPLA) and an agglomerate of around 8–10 µm is seen in Figure 1d after melt mixing. The agglomeration of the dispersed CNCs during melt mixing could be due to the de-sulfation of CNCs at higher temperatures [46]. Another contributing phenomenon may be the intrinsic poor affinity of CNCs with the polymer matrices. The Hansen solubility parameters (HSP) and the HSP distances of PLA, PBAT, CNCs, and DMF, and their relative energy differences (RED) were calculated at room and processing temperatures (detailed information is presented in SM). According to the calculated HSP distances between CNCs and the polymers compared to the HSP radius of CNC, (Table S1), at room and processing temperatures the RED is more than 1, which indeed represents a rather poor chemical affinity between CNCs and both polymers. This is in contrast to the high affinity of CNCs with DMF (RED < 1). As a result, the dispersed CNCs, which are in a metastable state after the removal of the solvent, may have a tendency to re-agglomerate during melt mixing. After solvent removal, in quiescent melt conditions, the high viscosity of the polymer matrices retards re-agglomeration since the Brownian motion is very slow [47]. However, in the internal mixer frequent CNC collisions may favor re-agglomeration. Our observations substantiate previous findings reported in the literature that while solution casting leads to a high level of dispersion and distribution, melt mixing following solution casting results in agglomeration of CNCs in the matrix [28,29,48]. These observations are in agreement with the rheological data presented in the next section.

#### 3.1.2. Rheology of Single Polymer Matrix Nanocomposites

**Figure 2:**
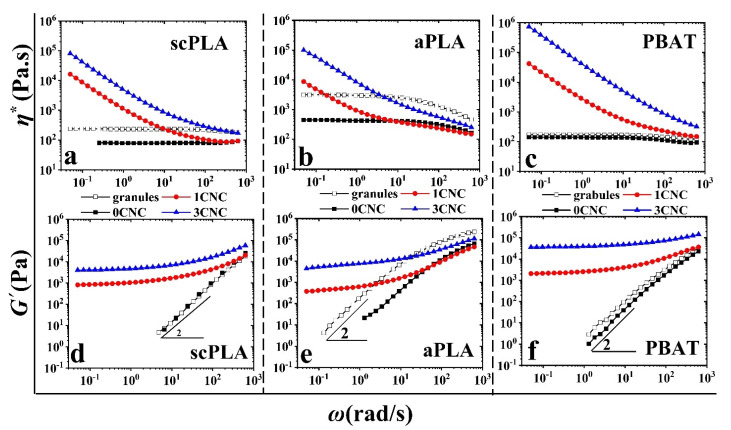
Complex viscosity (**a**–**c**) and storage modulus (**d**–**f**) of the neat polymers (0 CNC) and nanocomposites (1 and 3 CNC) prepared from solution casting (filled symbols) as functions of angular frequency and CNC content. Empty symbols are small amplitude oscillatory shear (SAOS) data of neat polymer samples prepared directly from granules using compression molding.

**Figure 3:**
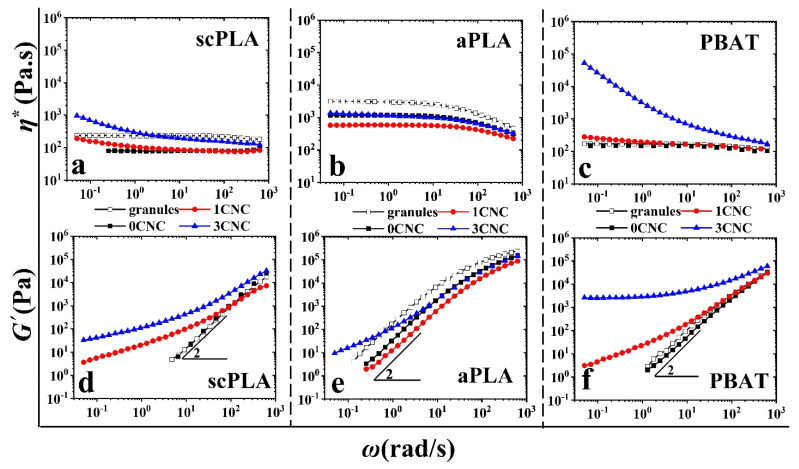
Effect of melt mixing (+IMM) on the complex viscosity (**a**–**c**) and storage modulus (**d**–**f**) of the neat polymers (0 CNC) and nanocomposites (1 and 3 CNC) prepared from solution casting +IMM (filled symbols) as functions of angular frequency and CNC content. Empty symbols are SAOS data of the neat polymer samples prepared directly from granules using compression molding.

Rheological analysis is another practical method to investigate the dispersion quality of nanoparticles in polymer nanocomposites [30,49,50]. Figure 2 and Figure 3 present the complex viscosity and storage modulus of the neat polymers and nanocomposites from solution casting (Figure 2) and the effect of melt mixing, +IMM, (Figure 3), respectively, as functions of angular frequency and CNC content. In Figure 2, the complex viscosities of the neat PLA (scPLA or aPLA) and PBAT exhibit a very broad Newtonian plateau at low frequencies. The storage modulus of the neat polymers also reveals a terminal zone with a slope of 2 at low frequencies, which is a characteristic of homogeneous molten polymers. For these samples prepared directly from solution casting, there are significant increases of the complex viscosity and storage modulus with the addition of CNCs (obviously more important for the 3 wt% CNC than for the 1 wt% CNC sample) as expected for the whole frequency range compared to the neat aPLA, scPLA and PBAT, also prepared from solution casting. What is more, the sudden upturn in the complex viscosity and the occurrence of a plateau in the storage modulus in the low frequency region for the 1 and 3 wt% CNC samples are characteristics of a network formation of the cellulose nanocrystals. These improvements in rheological properties are in accordance with the microscopic analysis of scPLA/1CNC nanocomposite sample prepared from solution casting (Figure 1a). We note that the relative increases in the rheological properties of the aPLA/CNC samples are less significant than for the scPLA/CNC nanocomposites, as expected for a lower degree of nanoparticle dispersion in the more viscous PLA (Figure 1b). We observe a decrease in the complex viscosity of both neat PLAs compared to the PLA prepared from the granules (empty squares in Figure 2 and Figure 3) mainly due to traces of solvent left in the samples after drying. For the neat PBAT, the effect of residual solvent is almost negligible, due to the crystallization of PBAT at the drying temperature (60–80 °C) that facilitated the solvent evaporation as explained in our previous work [30], in which more significant effects of residual solvents have been reported for the same polymers but using different solvents. The effect of the remaining solvent is also less visible on scPLA as compared to aPLA and this could be attributed to the higher viscosity of aPLA, which may hinder solvent removal during drying.

Figure 3 shows the effect of melt mixing (+IMM) on the complex viscosity and storage modulus of samples prepared from solution casting. When compared to Figure 2, there are considerable decreases of the SAOS properties due to the agglomeration of the CNCs and, although the addition of 1 and 3 wt% CNCs could slightly improve the complex viscosity and storage modulus of scPLA and PBAT, there is a decrease in the rheological properties of aPLA nanocomposites with respect to the neat aPLA. This is clearly seen in the 1 wt% sample, indicative of the degradation of the aPLA during melt mixing and possibly due to the presence of more remaining solvent in aPLA. A similar lack of rheological enhancements in SAOS have been reported for other polymer nanocomposites containing CNCs [28,51,52].

Overall, such a decrease in viscoelastic properties is a clear indication of the disruption of the CNC dispersion when the samples were melt blended in the internal mixer. In other words, as there was no CNC surface treatment or compatibilizer, the dispersed cellulose nanocrystals dramatically tended to re-agglomerate mostly due to the low chemical affinity of CNCs with both polymers and possible de-sulfation of CNCs at higher temperatures during the melting process, as discussed in the previous section. The SEM images of Figure 1b,d confirm the drastic effect of melt mixing on the CNC dispersion in the scPLA/1CNC and aPLA/1CNC nanocomposites, respectively.

**Figure 4:**
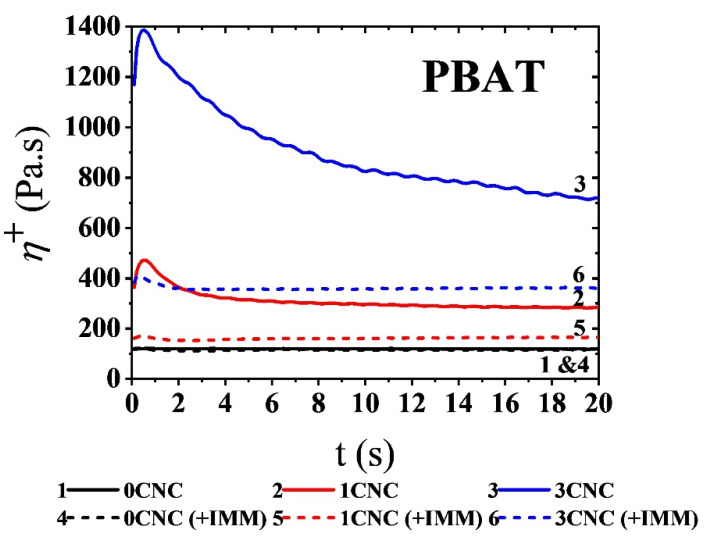
Variations of the shear stress growth coefficient, *η^+^,* of the PBAT/CNC nanocomposites as functions of time for an imposed shear rate of 5 s^−1^. Solid and dashed lines represent the samples prepared from solution casting and solution casting followed by melt mixing, respectively.

Figure 4 presents the stress growth coefficient, *η+*, of the neat PBAT and its nanocomposites containing 1 and 3 wt% CNCs in a stress growth (start-up) experiments at an imposed shear rate of 5 s^−1^ for the first 20 s of the test that lasted 480 s (*η+* was about constant for t ≥ 20 s). The solid and dash lines represent the PBAT/CNC nanocomposites prepared from solution casting followed or not by melt mixing, respectively. At this low applied shear rate, the neat PBAT does not show any overshoot for the sample before and after melt mixing as there is no network formed in absence of CNCs. On the other hand, the observation of overshoots (mainly in solution cast samples) in the transient viscosity versus time is assigned to the network of cellulose nanocrystals in the matrix of PBAT. Melt mixing (dashed lines in Figure 4) results in a severe decrease in the intensity of the overshoot due to the re-agglomeration of CNCs during melt mixing. For the higher concentration CNC sample, the overshoot also becomes larger, revealing a stronger CNC network. Bagheriasl et al. [26] showed similar behavior for the nanocomposites of PLA/CNC (same grade of scPLA in this paper) prepared from solution casting. Similar results were obtained for scPLA/CNC and aPLA/CNC nanocomposites and are presented in SM (Figure S3a,b). Due to the startup flow experiments, the CNC networks in scPLA, aPLA, and PBAT were destroyed and the rebuild-up of these networks was investigated through SAOS time sweep experiments for 1800 s and the result are presented in the SM (Figure S4). There is no structural build-up for all neat polymers before and after melt mixing, as expected. On the other hand, the structural build-up is clear for all single polymer matrix nanocomposites especially the ones from solution casting with a larger CNC content. SAOS frequency sweep tests were also conducted after stress growth experiments and the results are presented in SM (Figures S5 and S6 for the samples from solution casting and solution casting followed by melt mixing, respectively). The structural recovery after time sweep tests may not be completed and there are significant differences between SAOS data before and after stress growth experiments (mostly for solution cast samples).

### 3.2. PLA/PBAT Blend Nanocomposites

#### 3.2.1. Morphology of Blend Nanocomposites

Based on the values of the surface energies of PLA, PBAT, and CNC and related interfacial tensions (details are presented in SM) between the PLA/PBAT/CNC components (Table S2), the wetting coefficient (Equation (S6)) is calculated as 6.67 (i.e., ω ≫ 1), which predicts that the thermodynamic equilibrium localization of CNC particles should be in the PBAT phase. The interfacial tension between both PLA and PBAT was also obtained from the best fits of the linear viscoelastic data using the Palierne model (Equations (S9) and (S10)) of the neat blends prepared both from granules and from solution casting followed by melt mixing. The results are presented in Figure S2. The respective interfacial tensions were found to be 1.2 (aPLA/PBAT (granules)), 0.8 (scPLA/PBAT (granules)), 1.8 (aPLA/PBAT (+IMM)), and 1.3 mN/m (scPLA/PBAT (+IMM)). These values are quite different than those calculated by the harmonic-mean equation as explained in SM (Table S2 and Figure S2). The lower calculated interfacial tension for scPLA/PBAT compared to aPLA/PBAT confirms the better compatibility between the semicrystalline PLA and PBAT, as expected from the HSP parameters (details are presented in SM). The 50% increase in the interfacial tension for the samples prepared from solution casting followed by melt mixing could be due to the fact that the Palierne model predictions are not very sensitive as shown by the predictions using the interfacial tension obtained for the blends prepared from granules and given the dashed lines in Figure S2c,d. Overall, using the interfacial tensions (Figure S2) calculated from the best fits of the Palierne model predictions of the SAOS data, the wetting parameter is calculated to be between 0 and 1, which predicts that the localization of CNCs should be at the interface of the PLA and PBAT, in contrast to the localization in PBAT predicted from the thermodynamics analysis.

**Figure 5:**
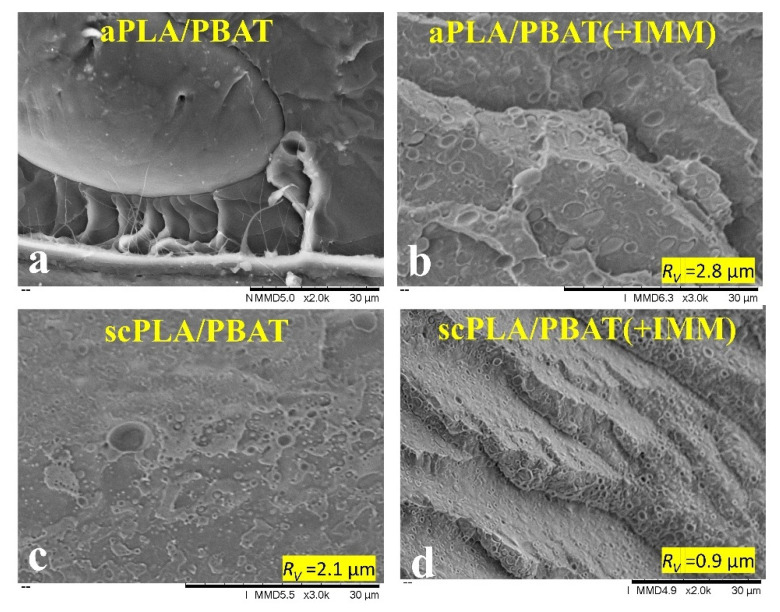
SEM images showing the morphologies of the neat blends from solution casting (**a**,**c**) and solution casting followed by melt mixing (**b**,**d**); (+IMM).

Figure 5 shows the SEM micrographs of cryo-fractured neat blends PLA (scPLA and aPLA)/PBAT prepared from solution casting (**a**,**c**) and solution casting followed by melt mixing (**b**,**d**). It is obvious that melt mixing has a substantial effect on the morphology of the neat blends, and the samples are more homogenous with finer morphologies. The volume average radius (*R_v_*) of the dispersed phase after melt mixing decreases from around 10–30 µm for aPLA/PBAT (observed from different SEM images at different locations) to 2.8 µm and from 2.1 to 0.9 µm for scPLA/PBAT, respectively. These decreases in the volume average radius of the dispersed phase after melt mixing are due to the higher deformation rate and better mixing via the internal mixer, compared to low mixing efficiency using a magnetic stirrer in solution casting. The finer morphology obtained for scPLA/PBAT is explained by the viscosity ratio closer to 1 [53] as can be deduced from Figure 2 and Figure 3. In most publications we examined, there was no clear attention paid to the difference between the morphology of blends from solution casting and melt mixing [54].

**Figure 6:**
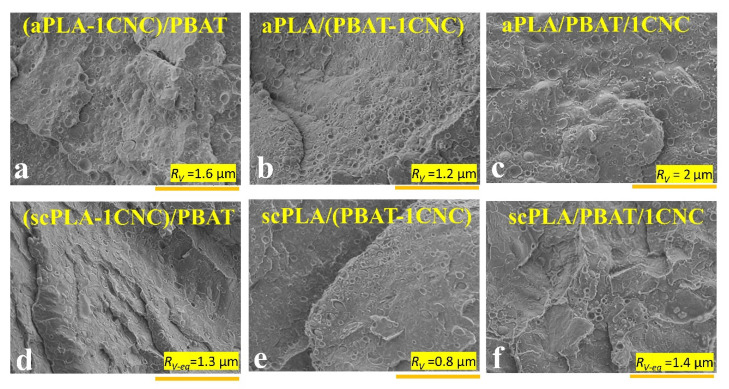
SEM images showing the morphologies of aPLA/PBAT/CNC (**a**–**c**) and scPLA/PBAT/CNC (**d**–**f**) blend nanocomposites. CNCs were initially (during the solution casting step) localized in the matrix (**a**,**d**), dispersed (**b**,**e**), and both phases (**c**,**f**). The scale bars are 30 µm.

Figure 6 presents the effect of the addition of CNCs on the morphology of PLA (scPLA and aPLA)/PBAT blends. It should be noted that the localization of the CNCs cannot be seen as the magnification level is too low. Adding CNCs to the aPLA/PBAT blend results in a decrease of the volume average radius of the dispersed phase no matter if the CNCs were initially localized in the matrix, dispersed, or both phases (Figure 6a–c). By adding CNCs to the aPLA/PBAT blend, *R_v_* decreases from 2.8 (Figure 5b) to 1.6, 1.2, and 2 µm for (aPLA-1CNC)/PBAT (Figure 6a), aPLA/(PBAT-1CNC) (Figure 6b), and aPLA/PBAT/1CNC (Figure 6c), respectively. The lowest *R_v_* is obtained when the CNCs were initially dispersed in PBAT (*η_PBAT_* < *η_PLA_*). Then, the CNCs in PBAT increased the viscosity of PBAT, which comes close to that of PLA, favoring the breakup of the dispersed droplets during mixing [55].

In the case of scPLA/PBAT, when the CNCs were initially localized in PBAT the *R_v_* values, 0.8 μm, (Figure 6e) are almost the same as the neat scPLA/PBAT, 0.9 μm, (Figure 5d). It is also worth mentioning that in the scPLA/PBAT/CNC blend nanocomposites the size of PBAT droplets varies between 0.5 to 5 µm, which shows a high polydispersity. In the other cases when CNCs were initially localized in the matrix or both phases, elongated PBAT droplets are observed and equivalent volume average radius, *R_v-eq_*, values of 1.3 and 1.4 µm are calculated for (scPLA-1CNC)/PBAT and scPLA/(PBAT-1CNC), respectively. It seems that the dispersed droplet-type morphology tends to be converted into a co-continuous one and this transformation could have a substantial effect on the final properties of the blend nanocomposites.

**Figure 7:**
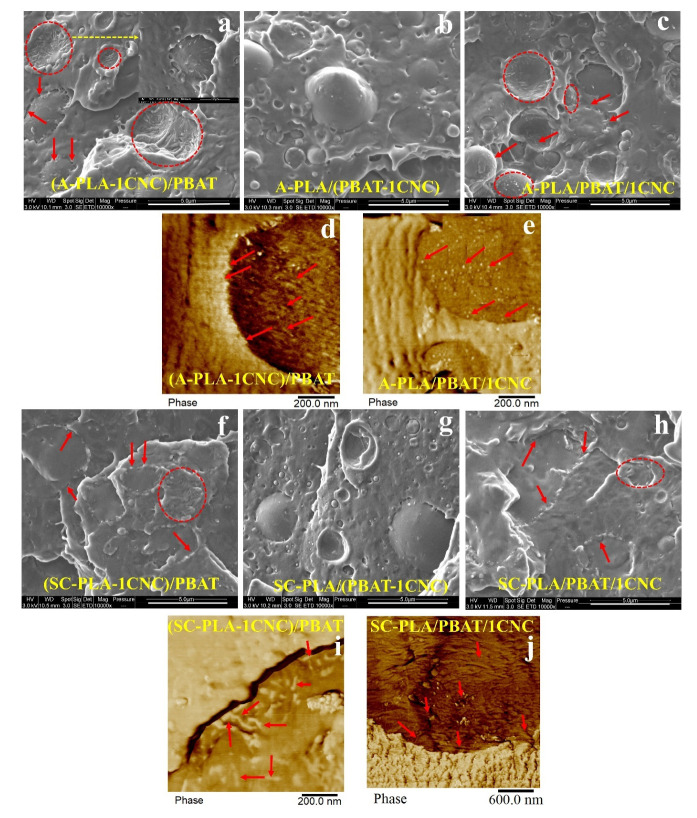
SEM (**a**–**c**,**f**–**h**) and AFM (**d**,**e**,**i**,**j**) images showing the localization of CNCs in the aPLA/PBAT/CNC (**a**–**e**) and scPLA/PBAT/CNC (**f**–**i**) blend nanocomposites. The CNCs were initially localized in the matrix (**a**,**d**,**f**,**i**), dispersed (**b**,**g**), and both phases (**c**,**e**,**h**,**i**) during the solution casting step.

To better localize the CNCs after melt mixing, SEM and AFM analyses were done at higher magnification and SEM and AFM phase images of aPLA/PBAT and scPLA/PBAT blend nanocomposites are presented in Figure 7a–j, respectively. As reported elsewhere [40,41], the CNCs particles appear as white dots (arrows) and also rods (circles) in these images. The cellulose nanocrystals in the aPLA/PBAT blend nanocomposites have migrated from the PLA phase, when CNCs were initially added to aPLA (Figure 7a,d) or both phases (Figure 7c,e), to the surface of the PBAT droplets (circles and arrows). This migration is clearer in the AFM images (Figure 7d,e) with a higher magnification. For the samples for which the CNCs were initially incorporated in the PBAT phase, it is difficult to tell from the SEM images if the CNCs are in the PBAT or aPLA phase, but as the thermodynamically favorable phase is PBAT the CNCs are most probably localized in the PBAT phase. For the scPLA/PBAT blend nanocomposites, it is difficult to identify the localization of CNCs through SEM images (Figure 7f–h). However, the AFM images (Figure 7i,j) clearly show the localization of CNCs at the interface of scPLA and PBAT droplets when the CNCs were initially added to the matrix or both phases. The CNCs are indicated by circles and arrows. All the findings in the SEM and AFM analyses are in accordance with the rheological properties which will be discussed in the next section.

#### 3.2.2. SAOS Behavior of PLA/PBAT/CNC Nanocomposites

**Figure 8:**
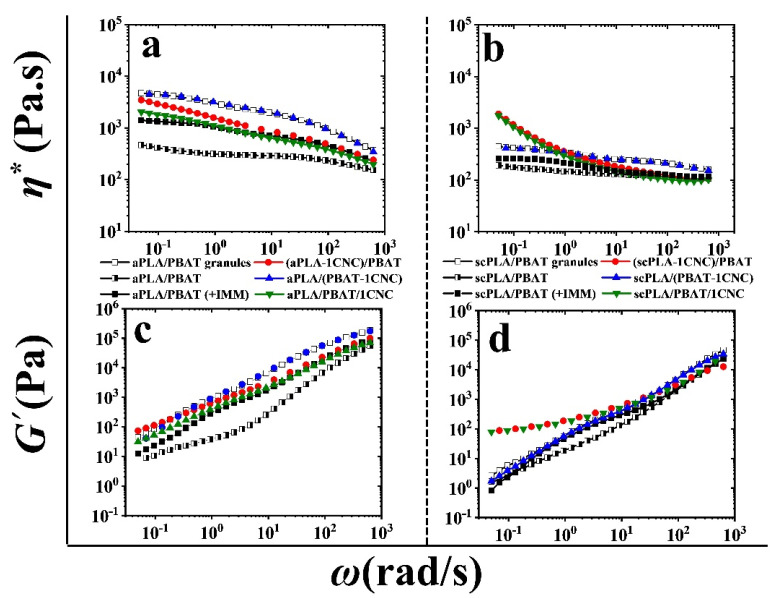
Complex viscosity (**a**,**b**) and storage modulus (**c**,**d**) versus angular frequency of aPLA or scPLA/PBAT/CNC blend nanocomposites. Empty, half filled-half empty, and filled black squares are for neat blends from granules, solution casting, and melt mixing of solution casted samples, respectively. Circles, upward, and downward triangles represent the blend nanocomposites when CNCs were initially localized in the matrix, dispersed, and both phases, respectively.

Figure 8 reports the complex viscosity (**a**,**b**) and storage modulus (**c**,**d**) of aPLA or scPLA/PBAT blends, from granules (empty squares), solution casting (half filled-half empty), and solution casting followed by melt mixing (filled squares), and their blend nanocomposites (circles, upward and downward triangles) after melt mixing. It is clear from Figure 8 that melt mixing (filled squares) increases the complex viscosity and storage modulus of the neat blend of aPLA or scPLA/PBAT prepared from solution casting (half filled-half empty squares). This is mainly because the morphology is finer (Figure 5b,d) and some residual solvent (DMF) has evaporated during melt mixing. However, it is still far from the complex viscosity of aPLA or scPLA/PBAT blends prepared from granules (empty squares), due to remaining solvent in the samples as discussed in a previous section. Adding CNCs to the PBAT during the solution casting step to prepare PLA/(PBAT-1CNC) blend nanocomposites results in an increase in the complex viscosity and storage modulus of the blend nanocomposites (upward triangles). These rheological results are in agreement with the SEM images of the blend nanocomposites when CNCs were introduced to the blends through PBAT; finer matrix-droplet morphologies are obtained, which in turn increase the rheological properties of the blend nanocomposites (Figure 6b,e). On the other hand, the addition of CNCs to the aPLA or scPLA during the solution casting step to prepare (PLA-1CNC)/PBAT (circles) and PLA/PBAT/1CNC (downward triangles) blend nanocomposites results in a slight increase and a sharp upturn in the complex viscosities at low frequencies of aPLA/PBAT/CNC and scPLA/PBAT/CNC blend nanocomposites, respectively (Figure 8a,b). Significant slope reductions in the storage modulus at low frequencies are observed mainly for scPLA/PBAT/CNC (Figure 8d). The SEM and AFM images of Figure 7 show that for (aPLA-1CNC)/PBAT and aPLA/PBAT/1CNC blend nanocomposites, there is a portion of cellulose nanocrystals that migrated to the thermodynamically stable phase (PBAT) and some CNCs are at the interface between the matrix and droplets. Moreover, in the scPLA/PBAT blend nanocomposites, when CNCs were initially incorporated into the matrix or in both phases, the complex viscosity results indicate a transition from a viscoelastic liquid to a solid behavior. This suggests that the CNCs form a 3D network in the blend, probably because enough CNC particles remain in the matrix. In the case of the (aPLA-1CNC)/PBAT blend nanocomposite, the observed finer morphology (Figure 8a) may explain the slightly larger values for the complex viscosity and storage modulus for that blend nanocomposite (Figure 8a,c). On the other hand, we see an almost identical rheological behavior for (scPLA-1CNC)/PBAT and scPLA/PBAT/1CNC (Figure 8b,d), in agreement with the SEM images of Figure 6d,f and Figure 7f,h, which show almost the same morphologies.

**Figure 9:**
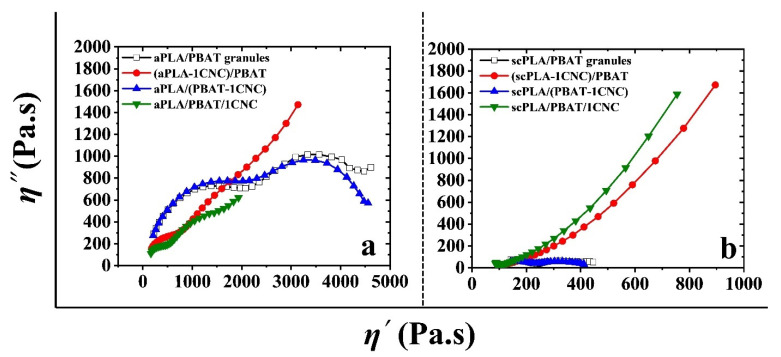
Cole-Cole plots of (**a**): aPLA/PBAT/CNC and (**b**): scPLA/PBAT/CNC blend nanocomposites.

The droplet relaxation phenomenon can be analyzed using plots of the imaginary component of the complex viscosity (*η″)* versus its real component (*η′*) in the form of Cole-Cole plots [56], as presented in Figure 9 for the PLA/PBAT/CNC blend nanocomposites. The left and right arcs in the Cole-Cole plots are the characteristics of the relaxation phenomena for the polymer chains and the droplets, respectively [56]. As seen from Figure 9, when CNCs were introduced to the nanocomposites through the PBAT phase (PLA/(PBAT-1CNC)), we have a matrix-droplet morphology with complete relaxation of the PBAT droplets. However, introducing 1 wt% of CNCs through PLA ((PLA-1CNC)/PBAT) or both phases (PLA/PBAT/1CNC) diminishes the arc of the Cole-Cole plots related to the relaxation of the dispersed phase and retards the relaxation of the droplets due to the network of CNCs formed through co-continuity of the phases or localization at the interface. Compared to the SEM images it could be concluded that the selective localization (at the interface) of CNCs in the PLA/PBAT/CNC blend nanocomposites retards the relaxation of PBAT droplets.

#### 3.2.3. Stress Growth Behavior and Coalescence

**Figure 10:**
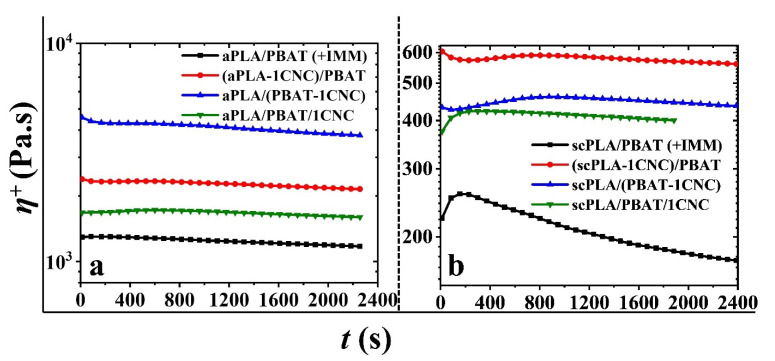
Stress growth coefficient (*η*^+^) as a function of time (*t*) for PLA/PBAT blends and PLA/PBAT/CNC blend nanocomposites containing 1 wt% CNCs; (**a**,**b**) are data for the amorphous and semicrystalline PLA blends, respectively.

Figure 10 presents the stress growth data for PLA/PBAT blends and PLA/PBAT/CNC blend nanocomposites containing 1 wt% CNCs. The experiments were carried out at 0.1 s^−1^ with a total shearing time of 2400 s. According to this figure, the more or less rapid decreases in the transient viscosity with time are an indication of coalescence of the PBAT droplets. In addition to coalescence, thermal degradation of PLA (mostly aPLA) could also contribute to the decrease in the transient viscosity over longer times. According to time-sweep experiments during 40 min, aPLA and scPLA showed a 10% drop in their transient viscosity within 20–25 min and around 35 min, respectively, while PBAT was stable. In the blend nanocomposites based on both PLAs, the transient viscosity drop is not as significant as those in the neat blends (Figure 10a,b). This decrease is clearer for the neat scPLA/PBAT in Figure 10b. This may be due to the viscosity ratio of the dispersed PBAT to the PLA matrix, which is around 1 and 0.1 in the scPLA/PBAT and aPLA/PBAT blends (Figure 3), respectively. The 10-fold larger viscosity ratio of the scPLA/PBAT blend could have a critical effect on more rapid coalescence.

**Figure 11:**
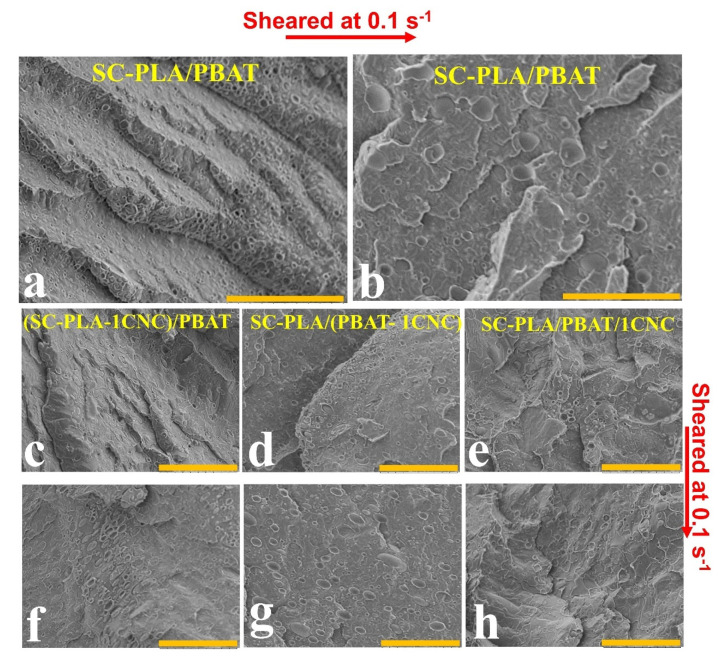
SEM images showing the dispersed PBAT phase in the scPLA matrix after (**a**) molding (i.e., non-sheared) and (**b**) sheared at a rate of 0.1 s^−1^ (**c**–**h**) the PBAT droplet morphological stability in presence of 1 wt% CNCs: (**c**–**e**) non-sheared and (**f**–**h**) sheared at a rate of 0.1 s^−1^. The scale bars are 30 µm.

In order to have a better understanding of the effect of shearing on the properties of the neat blends and their nanocomposites, the morphology of the neat scPLA/PBAT blend and scPLA/PBAT/CNC blend nanocomposites containing 1 wt% CNCs have been investigated and the results are shown in Figure 11 for samples before and after the stress growth experiments. As seen from Figure 11a,b, a significant droplet coalescence occurred for the neat scPLA/PBAT blend during shearing, and the volume average radius increases from 0.9 to 1.0–3.0 µm (Table 2). In contrast, Figure 11c–h show no or minor morphological changes after shearing for the blend nanocomposites, and no matter the initial localization of CNCs, the morphologies are uniform and, as presented in previous parts, the cellulose nanocrystals stayed in the dispersed phase or at the interface of the phases, before and after shearing (see Table 2). This suggests that the cellulose nanocrystals in the dispersed phase or at the interface between the two polymers served as a droplet coalescence barrier during shearing. To confirm the absence of coalescence in the PLA/PBAT nanocomposites, time sweep experiments were conducted at a frequency of 1 rad/s for 1 h, and the results are presented in the SM (Figure S7).

## 4. Conclusions

In this work, the localization of CNCs in PLA (amorphous and semicrystalline)/PBAT blends through solution casting and melt mixing methods and its effect on the rheology and morphology as well as on morphological stability under shear were studied in detail. PLA/CNC or PBAT/CNC neat nanocomposites obtained from solution casting exhibit a high level of CNC dispersion in each polymer. The effect of the melt mixing on the single polymer matrix nanocomposites was also investigated, showing a significant re-agglomeration of the CNCs. For preparing the blend nanocomposites, the CNCs were initially localized in the matrix, dispersed, or with both phases during the solution casting step, and the final localization of CNCs were studied after melt mixing. In most cases, it was shown that the incorporation of CNCs decreased the PBAT droplet size and created a finer morphology in the blend nanocomposites. When CNCs were initially dispersed, in PLA or both phases, they tended to be localized at the interface of the PLA and PBAT phases, which was favorable for stabilization of the blend morphology under shear flow. When CNCs were introduced to the blend nanocomposites through the PBAT phase, a matrix-droplet morphology was obtained with a complete relaxation of the PBAT droplets. However, introducing 1 wt% of CNCs, through PLA or both phases, retarded the relaxation of the droplets due to the network formation of CNCs. Applying a shear rate of 0.1 s^−1^ induced a pronounced droplet coalescence in the neat PLA/PBAT blend, whereas adding 1 wt % CNCs significantly prevented PBAT droplet coalescence. In this context, it could be noted that when solvents are used in the preparation method, the choice of solvent and the possibly remaining solvent in the prepared samples have a great effect on the rheological and morphological properties, but it should still be considered a proper method for the dispersion of unmodified CNCs in hydrophobic polymers.

## Figures and Tables

**Table 1:** Mixing sequences to prepare the blend nanocomposites and final composition.

Notation	Mixing Steps	Real Final Composition, wt% Poly (Lactic Acid)/ Poly (Butylene Adipate-Co-Terephthalate)/Cellulose Nanocrystals (PLA/PBAT/CNC)
PLA/PBAT granules	Mixing the neat PLA and PBAT granules using the internal mixer to prepare neat blends	75/25/0
PLA/PBAT	Mixing the neat PLA and PBAT granules using the solution casting to prepare neat blends	75/25/0
PLA/PBAT (+IMM)	Mixing the neat PLA and PBAT from solution casting followed by melt mixing via the internal mixer to prepare neat blends	75/25/0
(PLA-1CNC)/PBAT (Mixing strategy 1)	Mixing PLA/1.4CNC with PBAT granules via the internal mixer. CNCs were initially mixed with PLA	74.95/25/1.05
PLA/(PBAT-1CNC) (Mixing strategy 2)	Mixing PBAT/4CNC with PLA granules via the internal mixer CNCs were initially mixed with the PBAT	75/24/1.0
PLA/PBAT/1CNC (Mixing strategy 3)	Mixing PLA/1CNC and PBAT/1CNC. via the internal mixer CNCs were initially mixed with both PLA and PBAT	74.25/24.75/1.0

**Table 2:** Volume average or equivalent average of PBAT droplet radius, *R_v_*, before and after shearing at a rate of 0.1 s^−1^ during 2400 s.

	Non-Sheared, *R_v_* or *R_v-eq_*	Sheared at 0.1 s^−1^, *R_v_* or *R_v-eq_*
scPLA/PBAT	0.90 µm	1.0–3.0 µm
(scPLA-1CNC)/PBAT	PBAT droplets are slightly elongated. 1.3 µm	PBAT droplets are slightly elongated. 1.4 µm
scPLA/(PBAT-1CNC)	0.8	0.8 µm
scPLA/PBAT/1CNC	PBAT droplets are slightly elongated. 1.4 µm	PBAT droplets are slightly elongated. 1.4 µm

## Data Availability

Not applicable.

## Supplementary Materials

The following are available online at https://www.mdpi.com/article/10.3390/nano11040857/s1, Figure S1. Mixing sequences to prepare the blend nanocomposites. (a) and (b) granules of the neat complementary polymers (PLA and PBAT granules) were added to the neat polymer matrix nanocomposites and (c) PLA and PBAT nanocomposites prepared from solution casting were melt mixed in the internal mixer. All single polymer matrix nanocomposites prepared initially from solution casting, Figure S2. Palierne model predictions; solid lines: best fits of $G^{\prime }$ and $G^{\prime \prime }$ for the blends of aPLA/PBAT and scPLA/PBAT prepared from granules (a,b) and solution casting followed by melt mixing (c,d) and dashed lines: comparison with the data of the 75/25 (wt%) aPLA/PBAT (c) and scPLA/PBAT (d) blends using the interfacial tension obtained from the best fits of the neat blends from granules (a,b), Figure S3. Variations of the shear stress growth coefficient, *η^+^,* with time, *t*, for scPLA/CNC (a) and aPLA/CNC (b) nanocomposites for an imposed shear rate of 5 s^−1^. The solid and dashed lines represent the samples prepared from solution casting and solution casting followed by melt mixing, respectively, Figure S4. Structure evolution expressed by the storage modulus versus time for scPLA/CNC (a), aPLA/CNC (b), and PBAT/CNC (c) nanocomposites right after the cessation of shear flow. Solid lines are the data of samples from solution casting and dashed lines represent the effect of melt mixing, Figure S5. Complex viscosity (a–c) and storage modulus (d–f) versus angular frequency of the neat polymers (0 CNC) and nanocomposites (1 and 3 CNC) from solution casting. Filled and empty symbols are SAOS data before and after stress growth experiments (sh), respectively, Figure S6. Effect of melt mixing (solvent casting+IMM) on the complex viscosity (a–c) and storage modulus (d–f) of the neat polymers (0 CNC) and nanocomposites (1 and 3 CNC) prepared through solution casting as functions of angular frequency and CNC content. Filled and empty symbols are SAOS data before and after stress growth experiments (sh), respectively, Figure S7. Complex viscosity (*η*)* versus time (*t*) of the neat PLA/PBAT (a: amorphous and b: semicrystalline) and blend nanocomposites reinforced with 1 wt% CNCs during 1 h at a frequency of 1 rad/s and strain amplitude of 0.001. Table S1. HSP distances and relative energy differences (RED) between PLA, PBAT, CNCs, and DMF, Table S2. Surface energy values of PLA, PBAT, and CNCs as well as the calculated interfa-cial tensions between CNCs, PLA, and PBAT at 180 °C.
