# Supplementary material for: Morphological and Rheological Properties of PLA, PBAT, and PLA/PBAT Blend Nanocomposites Containing CNCs
Source: Nanomaterials (Basel). 2021 Mar 27;11(4):857. doi: 10.3390/nano11040857 (PMC8065413; doi:10.3390/nano11040857)
Supplement: Supplementary file 1 [file nanomaterials-11-00857-s001.pdf]

nanomaterials

Supplementary Materials

Morphological and Rheological Properties of PLA, PBAT, and PLA/PBAT Blend Nanocomposites Containing CNCs

Mojtaba Mohammadi [1], Marie-Claude Heuzey [1], Pierre J. Carreau [1,*] and Aurélie Taguet [2]

[1] Center for High Performance Polymer and Composite systems (CREPEC), Department of Chemical Engineering, École Polytechnique de Montréal, Montreal, QC H3T 1J4, Canada; mojtaba.mohammadi@polymtl.ca (M.M.); marie-claude.heuzey@polymtl.ca (M.-C.H.); pierre.carreau@polymtl.ca (P.J.C.)

[2] Polymers Composites and Hybrids (PCH), IMT Mines Ales, 30319 Ales, France; aurelie.taguet@mines-ales.fr (A.T.)

* Correspondence: pierre.carreau@polymtl.ca

Single polymer matrix and blend nanocomposites preparation

The aPLA, scPLA, and PBAT were first dried in a vacuum oven overnight at 55 °C and then each polymer (about 40 g) was dissolved in 70 mL DMF using a magnetic stirrer for 2 h at 70 °C until complete dissolution. Separately, the desired amount of CNCs (between 0.4–1.6 g based on the final weight percentage of CNCs in the neat and blend nanocomposites) was dispersed in 70 mL of DMF using a water bath sonicator (FS30 100 Watts Ultrasonic Cleaner, Fisher Scientific, Pittsburg, PA, USA) for 2 h at room temperature. Afterward, the neat polymer solution was added to the CNC suspension and magnetic stirring was continued for another 2 h at 70 °C to ensure a good distribution and dispersion of the nanoparticles despite the rather high viscosity of the solution. Then, the mixtures were poured into a petri dish and dried in an oven in two steps. First, the samples were put in the vacuum oven (0.9 bar) with air circulation set at 60 °C for 2 days. Then, the drying process was completed for another 2 days at 80 °C under vacuum (−0.65 bar). After removing the samples, the nanocomposites containing the low molecular weight PLA (scPLA) could be ground into powder using a coffee grinder, but the high molecular weight PLA (aPLA) and PBAT samples had to be chopped to very small pieces using scissors.

Figure S1 shows blend nanocomposites preparation containing 75 wt% PLA and 25 wt % PBAT and overall, 1 wt % CNCs. In the first two mixing strategies (Figures S1a,b), granules of the neat complementary polymer (dried overnight at 55 °C) were added to the single polymer matrix nanocomposites in the internal mixer. In the third strategy (Figure S1c) both PLA and PBAT nanocomposites containing 1 wt% prepared from solution casting were melt mixed in the internal mixer.

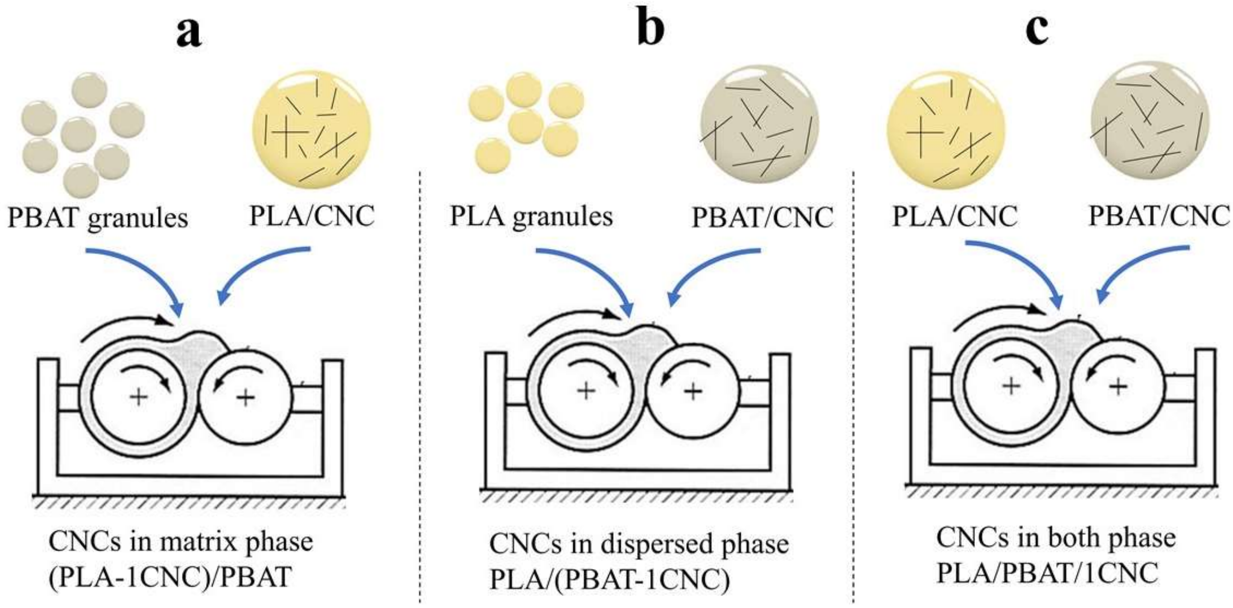

Figure S1. Mixing sequences to prepare the blend nanocomposites. (**a**) and (**b**) granules of the neat complementary polymers (PLA and PBAT granules) were added to the neat polymer matrix nanocomposites and (**c**) PLA and PBAT nanocomposites prepared from solution casting were melt mixed in the internal mixer. All single polymer matrix nanocomposites prepared initially from solution casting.

Hansen solubility parameters (HSP) for PLA, PBAT, and CNCs

In our research group, the Hansen solubility parameter (HSP) theory [1] was used to determine the chemical affinity of CNCs [2,3] and predict both their level of colloidal stability and behavior upon solvent casting [4,5]. The HSP theory is based on cohesive energy density. The total cohesion parameter, δ_T, is the square root of the cohesive energy density and is split into three components, dispersive (δ_D), dipole-dipole (δ_P) and hydrogen-bonding (δ_H) (and other Lewis acid/base) interactions. δ_T is expressed as follows, with units of MPa$^{1/2}$:

$$\delta_T^2 = \delta_D^2 + \delta_P^2 + \delta_H^2 \quad \text{(S1)}$$

The chemical distance $R_{a,\text{A-B}}$ between two substances A {$\delta_{D,A}$; $\delta_{P,A}$; $\delta_{H,A}$} and B {$\delta_{D,B}$; $\delta_{P,B}$; $\delta_{H,B}$} may then be expressed as the norm of the $vector\ \overrightarrow{AB}$ in the HSP graph:

$$R_{a,\text{A-B}} = \|\overrightarrow{AB}\| = \sqrt[2]{4(\delta_{D,A}-\delta_{D,B})^2 + (\delta_{P,A}-\delta_{P,B})^2 + (\delta_{H,A}-\delta_{H,B})^2} \quad \text{(S2)}$$

Literature reports HSP values at room temperature of ~{18.1; 20.4; 15.3} MPa$^{1/2}$ [3], ~ {18.5; 8.0; 7.0} MPa$^{1/2}$ [6], and ~ {18.0; 5.6; 8.4} MPa$^{1/2}$ [6] for CNCs, PLA, and PBAT, respectively, along with solvent solubility radii $R_{0,\text{CNC}}$ = 7.8 MPa$^{1/2}$, $R_{0,\text{PLA}}$ ≈ 8 MPa$^{1/2}$, and $R_{0,\text{PBAT}}$ ≈ 4.5 MPa$^{1/2}$ [3,4,6]. R_0 is the critical threshold chemical distance for the substance to be dispersed or dissolved in a solvent. Solvents whose chemical distances with CNCs are smaller than 7.8 MPa$^{1/2}$ were found to adsorb significantly on CNC surfaces [3]. Therefore, by defining a sphere of radius R_0, which contains all the good solvents, we can identify a relative energy difference, RED = R_a/R_0. Solvents with RED ≤ 1 are considered as good suspending media. Also, RED ≤ 1 between two materials indicates a good chemical affinity.

A combination of electrostatic and solvation-induced stabilization was found to be necessary to reach sufficient colloidal stability for CNC particles [4] and among the best-suspending media, dimethylformamide (DMF), with HSP values of ~{17.4; 13.7; 11.3} MPa$^{1/2}$ stands after water, formamide, N-methylformamide, and dimethylsulfoxide (DMSO) [7].

If the temperature rises, then the density decreases and as a result, the HSP values decrease. The effect depends on ΔT, (the change of temperature with respect to 25 °C), and the thermal expansion coefficient, α, which is taken to be 0.0007/K for polymers, CNCs, and DMF [8]. So, dispersive, dipole-dipole, and hydrogen-bonding (and other Lewis acid/base) interactions in the solubility parameters will change as follows [8]:

$$\delta_D^T = \delta_D \cdot (1 - \Delta T \cdot \alpha \cdot 1.25) \tag{S3}$$

$$\delta_P^T = \delta_P \cdot (1 - \Delta T \cdot \alpha/2) \tag{S4}$$

$$\delta_H^T = \delta_H \cdot (1 - \Delta T(0.00122 + \alpha/2) \tag{S5}$$

According to Equations S3, S4, and S5, the HSP values at 180 °C decrease to ~{15.6; 19.2; 11.4} $MPa^{1/2}$ for CNCs, ~{15.9; 7.5; 5.3} $MPa^{1/2}$ for PLA, ~{15.5; 5.3; 6.3} $MPa^{1/2}$ for PBAT, and ~{15.0; 12.9; 8.6} $MPa^{1/2}$ for DMF. Also, Table S1 reports the HSP distances and relative energy differences (RED) between PLA, PBAT, CNCs, and DMF (Equation S2) at 25 and 180 °C.

Table S1. HSP distances and relative energy differences (RED) between PLA, PBAT, CNCs, and DMF.

	25 °C / RED	180 °C / RED
$R_{a,\mathrm{PLA\text{-}PBAT}}$	3.0 $MPa^{1/2}$/ ≤1	2.6 $MPa^{1/2}$/ ≤1
$R_{a,\mathrm{CNC\text{-}DMF}}$	7.9 $MPa^{1/2}$/ ≤1	6.9 $MPa^{1/2}$/ ≤1
$R_{a,\mathrm{CNC\text{-}PLA}}$	14.9 $MPa^{1/2}$/ >1	13.2 $MPa^{1/2}$/ >1
$R_{a,\mathrm{CNC\text{-}PBAT}}$	16.3 $MPa^{1/2}$/ >1	14.8 $MPa^{1/2}$/ >1

According to the HSP distances reported in Table S1, the RED values for $R_{a,\mathrm{CNC\text{-}PLA}}$ and $R_{a,\mathrm{CNC\text{-}PBAT}}$ compared to the HSP radius of CNCs, $R_{0,\mathrm{CNC}}$ = 7.8 $MPa^{1/2}$, are greater than 1 at 25 and 180 °C and, hence, they predict a poor chemical affinity between CNCs and both polymers. In contrast, the RED is less or equal to 1 for PLA and PBAT, and CNCs and DMF. This highlights a good chemical affinity between PLA and PBAT, and CNCs and DMF. These results are consistent with the difficulties that have been reported to disperse unmodified CNCs in these matrices [9,10]. Also, it should be mentioned that the HSP parameters are affected by molecular weight and crystallinity [6,11]. For the same two polymers of different molecular weights, the HSP radius of low molecular weight is larger than the high molecular weight [12]. Hence, scPLA with a low molecular weight and higher crystallinity compared to aPLA should have a larger HSP radius, R_0. As a result, the RED for scPLA and PBAT with larger R_0 is smaller than that for aPLA and PBAT. So, the chemical affinity between scPLA and PBAT with smaller RED is better than that for aPLA and PBAT, although they are phase separated.

Surface energy and interfacial tension

The Young model can predict the localization of solid particles in polymer blends [13] based on the wetting parameter, ω_a, defined by :

$$\omega_a = \frac{\gamma_{1s} - \gamma_{2s}}{\gamma_{12}} \tag{S6}$$

where γ_{1s}, γ_{2s}, and γ_{12} are the interfacial tensions between polymer 1 and solid particles, polymer 2 and solid particles, and polymers 1 and 2, respectively. Thermodynamically, the particles would be localized in phase 2 when $\omega_a > 1$, while phase 1 is the preferred location of the solid particles when $\omega_a < -1$. The solid particles will be thermodynamically localized at the interface when $-1 \leq \omega_a \leq 1$ [13].

The harmonic-mean approach is used to estimate the interfacial tension between PLA and PBAT [14]:

$$\gamma_{ij} = \gamma_i + \gamma_j - 4\left(\frac{\gamma_i^d \gamma_j^d}{\gamma_i^d + \gamma_j^d} + \frac{\gamma_i^p \gamma_j^p}{\gamma_i^p + \gamma_j^p}\right) \tag{S7}$$

and the interfacial tension between PLA and CNC, and PBAT and CNC is determined via the geometric-mean equation [14]:

$$\gamma_{ij} = \gamma_i + \gamma_j - 2\left[\sqrt{\gamma_i^d \gamma_j^d} + \sqrt{\gamma_i^p \gamma_j^p}\right] \tag{S8}$$

where γ_{ij} is the interfacial tension between components i and j, γ_i is the surface tension of material *i* and γ_i^d and γ_i^p are the dispersive and polar components, respectively, of the surface tension of the same material. The harmonic mean approach is more accurate for estimating the interfacial tensions between low surface energy materials while the geometric mean equation can predict the interfacial tensions between low and high surface energy materials more accurately [14]. We can obtain the values of the interfacial tensions between the PLA/PBAT/CNC components and the wetting coefficient to estimate the localization preference of CNCs within the blend. The interfacial tensions were calculated based on surface tension values for PLA, PBAT, and CNCs at 25 °C reported in the literature [15–17]. To obtain the surface tension of the polymer components at the processing temperature (180 °C), a temperature coefficient of 0.06 mJ·m^{-2}·K^{-1} was used to extrapolate the surface tension values at 25 °C [18]. Also, the CNC surface tension was estimated at 180 °C using a temperature coefficient of −0.2 mJ·m^{-2}·K^{-1} reported in the literature [17]. The surface tension parameters of the blend nanocomposite components at the processing temperature of 180 °C and their estimated interfacial tensions are reported in Table S2. Considering PLA as phase 1 and PBAT as phase 2 and replacing the estimated interfacial tensions in Equation S6, the wetting parameter is calculated as 6.67 (i.e., ω ≫ 1), which predicts that the thermodynamic equilibrium localization of CNCs should be in the PBAT phase.

Table 2. Surface energy values of PLA, PBAT, and CNCs as well as the calculated interfacial tensions between CNCs, PLA, and PBAT at 180 °C.

	**At 25 °C**			**At 180 °C**			**Interfacial tension at 180 °C γ_{12} (mN/m)**		
	**γ (mN/m)**	**γ^d (mN/m)**	**γ^p (mN/m)**	**γ (mN/m)**	**γ^d (mN/m)**	γ^p (mN/m)	**PLA**	**PBAT**	**CNC**
PLA	39.4	33.6	5.8	30.1	25.7	4.4	-	0.06 [a]	3.4 [b]
PBAT	38.4	32.1	6.3	29.1	24.3	4.8	0.06 [a]	-	3.0 [b]
CNCs	68.9	40.9	28	37.9	22.5	15.4	3.4 [b]	3.0 [b]	-

[a] Calculated from the harmonic-mean approach (Equation S7)

[b] Calculated from the geometric-mean approach (Equation S8)

Also, in this work, the emulsion model of Palierne [19] was used to determine the interfacial tension of PLA and PBAT from the SAOS data [19,20]. This model (Equations S9 & S10) is used for the neat blends prepared from granules and solution casting followed by melt mixing with narrow droplet size distribution. As the average droplet size for the neat blends prepared by solution casting are more than 2 μm with varying droplet size distribution and coarse morphology, the Palierne model is not applicable for the interfacial analysis of those neat blends [21]. The complex modulus of a blend of narrow droplet size distribution ($R_v/R_n \leq 2$, where R_n is the number-average diameter) and constant interfacial tension is expressed by [21]:

$$G_b^*(\omega) = G_m^*(\omega)\frac{1+3\phi H^*(\omega)}{1-2\phi H^*(\omega)} \tag{S9}$$

and

$$H^*(\omega) = \frac{4\left(\frac{\gamma_{12}}{R_v}\right)[2G_m^*(\omega)+5G_d^*(\omega)]+[G_d^*(\omega)-G_m^*(\omega)][16G_m^*(\omega)+19G_d^*(\omega)]}{40\left(\frac{\gamma_{12}}{R_v}\right)[G_m^*(\omega)+G_d^*(\omega)]+[2G_d^*(\omega)+3G_m^*(\omega)][16G_m^*(\omega)+19G_d^*(\omega)]} \tag{S10}$$

where ϕ, ω, and γ_{12} are the volume fraction of droplets of volume average radius, R_v, the angular frequency, and the interfacial tension, respectively. $G_b^*(\omega)$, $G_m^*(\omega)$, and $G_d^*(\omega)$ are the complex

modulus of the blend, matrix, and dispersed phase, respectively. The interfacial tension was obtained by fitting the data to the model predictions for the neat blends (Figures S2a–d) using MATLAB (MATLAB software package R2019b, the Mathworks, Inc. MA, USA) and for the values of R_v determined from the SEM images. The storage and loss moduli of the blends can be expressed explicitly in terms of the moduli of both components [21,22].

$$G_b' = \frac{1}{D}[G_m'(B_1B_2 + B_3B_4) - G_m''(B_4B_1 - B_2B_3)] \tag{S11}$$

$$G_b'' = \frac{1}{D}[G_m'(B_1B_4 - B_2B_3) + G_m''(B_1B_2 + B_3B_4)] \tag{S12}$$

where the constants are expressed by:

$$B_1 = C_1 - 2\phi C_3 \tag{S13}$$

$$B_2 = C_1 + 3\phi C_3 \tag{S14}$$

$$B_3 = C_2 - 2\phi C_4 \tag{S15}$$

$$B_4 = C_2 + 3\phi C_4 \tag{S16}$$

$$D = (C_2 - 2\phi C_4)^2 + (C_1 - 2\phi C_3)^2 \tag{S17}$$

with

$$C_1 = 40\left(\frac{\gamma_{12}}{R_v}\right)(G_m' + G_d') + 38\left(G_d'^2 - G_d''^2\right) + 48\left(G_m'^2 - G_m''^2\right) + 89(G_m'G_d' - G_m''G_d'') \tag{S18}$$

$$C_2 = 40\left(\frac{\gamma_{12}}{R_v}\right)(G_m'' + G_d'') + 98G_m'G_m'' + 76G_d'G_d'' + 89(G_m''G_d' - G_m'G_d'') \tag{S19}$$

$$C_3 = 4\left(\frac{\gamma_{12}}{R_v}\right)(2G_m' + 5G_d') - 16\left(G_m'^2 - G_m''^2\right) + 19\left(G_d'^2 - G_d''^2\right) - 3(G_m'G_d' - G_m''G_d'') \tag{S20}$$

$$C_4 = 4\left(\frac{\gamma_{12}}{R_v}\right)(2G_m'' + 5G_d'') - 32G_m'G_m'' + 38G_d'G_d'' - 3(G_m''G_d' + G_m'G_d'') \tag{S21}$$

Figure S2 shows that the best fits (a–d; solid lines) are quite adequate, and the interfacial tensions were found to be 1.2 mN/m (aPLA/PBAT granules), 0.8 mN/m (scPLA/PBAT granules), 1.8 mN/m (aPLA/PBAT (+IMM)), and 1.3 mN/m (scPLA/PBAT (+IMM)). These values are quite different than those estimated from the harmonic-mean equation. The lower calculated interfacial tension for scPLA/PBAT compared to aPLA/PBAT confirms the better compatibility between scPLA and PBAT. This better compatibility is expected from the HSP parameters as explained above. Also, the increase in interfacial tension for the samples prepared from solution casting followed by melt mixing could be due to fact that the Palierne model predictions are not always very sensitive to the interfacial tension as shown by Lacroix et al. [22] and demonstrated here by the predictions using the interfacial tension obtained for the blends prepared from granules, given the dashed lines in Figure S2c & d. For both blends, the fits appear to be as good and one may assume that the interfacial tension values obtained for the blends prepared from granules are quite reasonable. Overall, using these interfacial tensions, the wetting parameter is calculated to be between 0 and 1, which predicts that the localization of CNCs should be at the interface of the PLA and PBAT, in contrast to the localization in PBAT predicted from the thermodynamics analysis presented above.

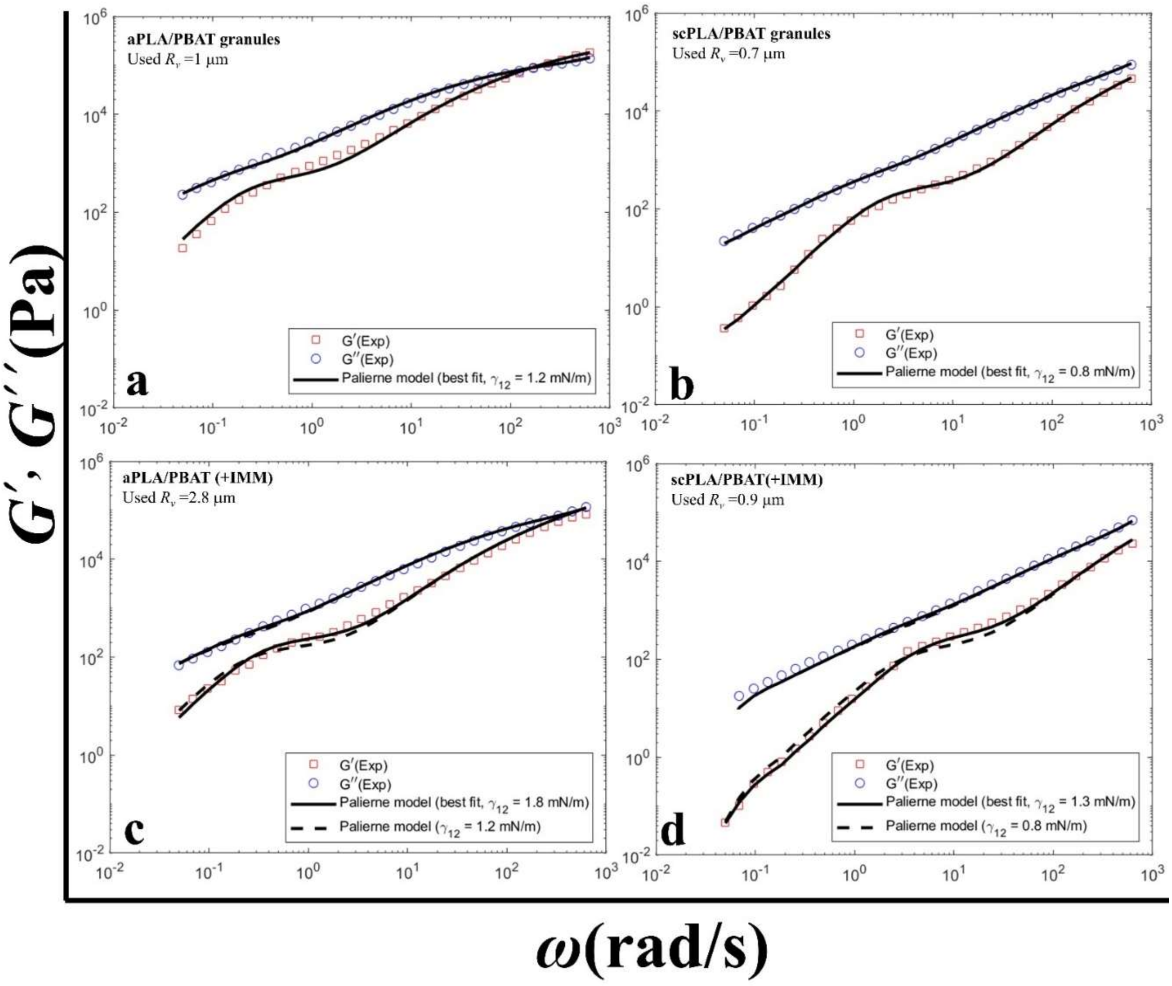

Figure S2. Palierne model predictions; solid lines: best fits of G' and G'' for the blends of aPLA/PBAT and scPLA/PBAT prepared from granules (**a** & **b**) and solution casting followed by melt mixing (**c** & **d**) and dashed lines: comparison with the data of the 75/25 (wt%) aPLA/PBAT (**c**) and scPLA/PBAT (**d**) blends using the interfacial tension obtained from the best fits of the neat blends from granules (**a** & **b**).

Additional rheological data of the single polymer matrix and blend nanocomposites

Figure S3 presents the stress growth coefficient, η^+, versus time, t, for scPLA/CNC (Figure S3a) and aPLA/CNC (Figure S3b) nanocomposites for an imposed shear rate 5 s^{-1} for the first 20 s of the test that lasted 480 s (η^+ was about constant for a time longer than 20 s). Solid and dashed lines represent the data for the samples prepared from solution casting and solution casting followed by melt mixing, respectively. Neat scPLA and aPLA do not show any overshoot before and after melt mixing in the absence of CNCs and network formation. On the other hand, the formation of a CNCs network in the matrix of both PLA results in significant overshoots mainly for solution cast samples. Also, melt mixing (dashed lines) results in a severe decrease in the intensity of overshoot due to the re-agglomeration of CNCs during melt mixing.

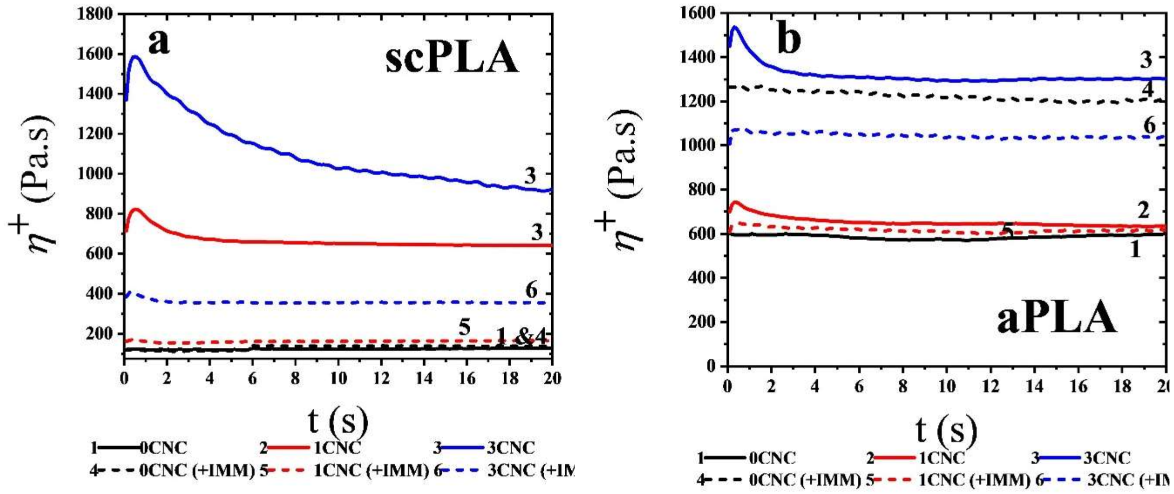

(**a**) (**b**)

Figure S3. Variations of the shear stress growth coefficient, η^+, with time, t, for scPLA/CNC (**a**) and aPLA/CNC (**b**) nanocomposites for an imposed shear rate of 5 s^{-1}. The solid and dashed lines represent the samples prepared from solution casting and solution casting followed by melt mixing, respectively.

Due to the startup flow experiments, the CNC networks in scPLA, aPLA, and PBAT were destroyed and the rebuild-up of the networks was investigated through SAOS time sweep experiments for 1800 s. Figure S4 reports the storage modulus versus time as solid and dashed lines for the single polymer matrix nanocomposites prepared from solution casting without melt mixer and followed by melt mixing, respectively. There is no structural build-up for all neat polymers before and after melt mixing, as expected. On the other hand, the structural build-up is clear for all single polymer matrix nanocomposites, especially the ones from solution casting with a larger CNC content. We note that after 1800 s, G' is still evolving as the structure has not attained an equilibrium value. The structural build-up can be affected by both the pre-shear rate, time of the startup flow experiments, and the concentration of CNCs.

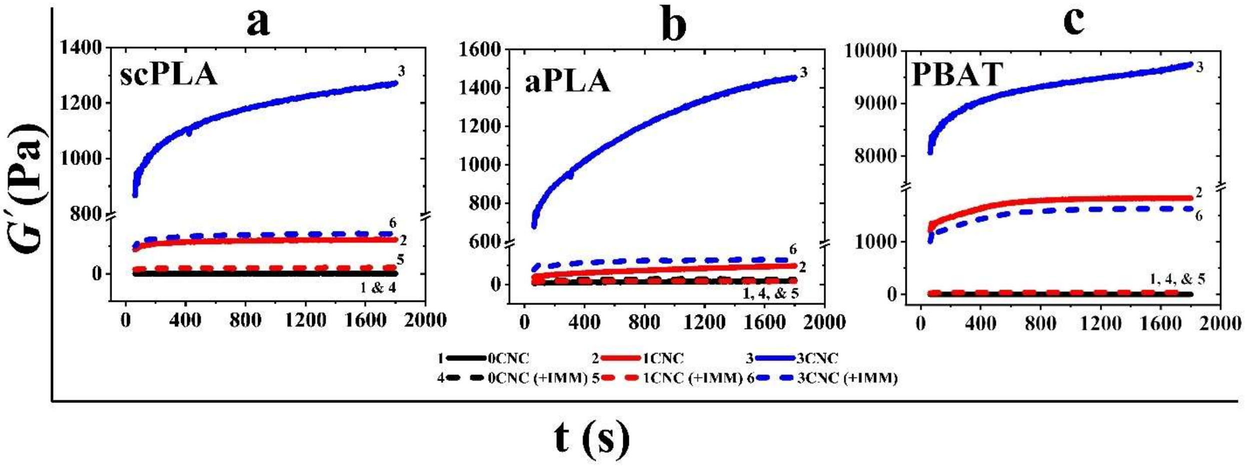

Figure S4. Structure evolution expressed by the storage modulus versus time for scPLA/CNC (**a**), aPLA/CNC (**b**), and PBAT/CNC (**c**) nanocomposites right after the cessation of shear flow. Solid lines are the data of samples from solution casting and dashed lines represent the effect of melt mixing.

The frequency sweep tests were conducted after the stress growth experiments of Figure S3 and the results are presented in Figures S5 and S6 for the samples from solution casting and solution casting followed by melt mixing, respectively. The reductions of the complex viscosity and storage

modulus for the samples from solution casting are larger than the ones after melt mixing. Also, the small decrease in the complex viscosity of aPLA/CNC nanocomposites at high frequencies compared to the sample prepared from granules (Figures 2 and 3 in the main manuscript) could be due to degradation of aPLA in the presence of CNCs. Although the structural recovery after time sweep tests may not be completed, these differences between solution casting and melt mixing could be due to the evaporation of the remaining solvent during melt mixing.

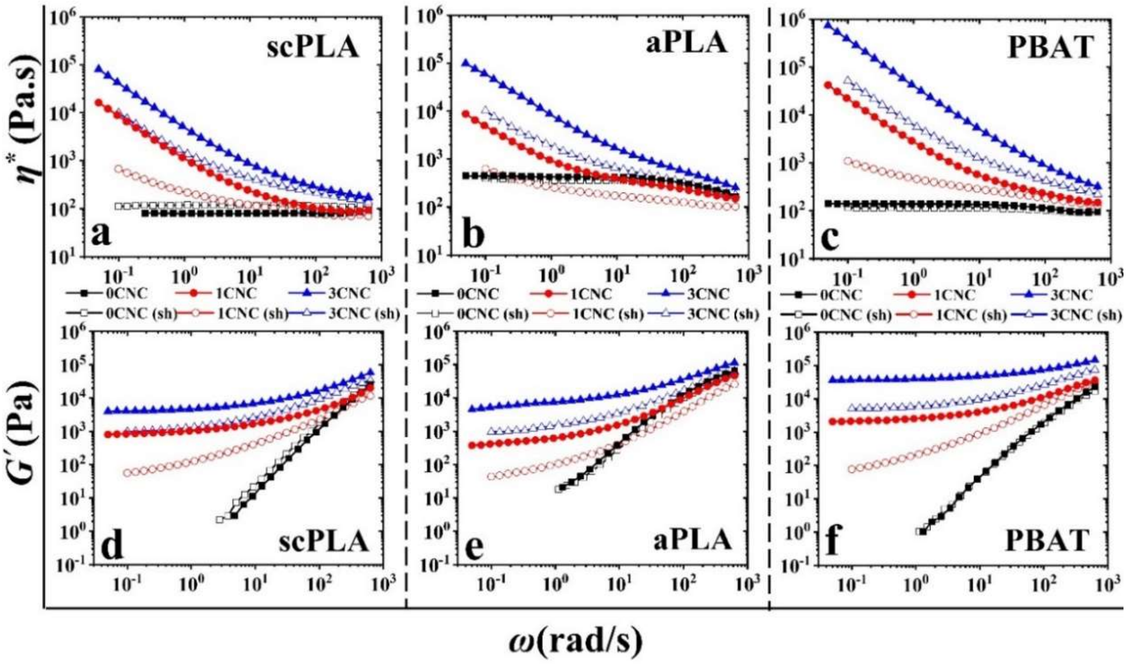

Figure S5. Complex viscosity (**a**-**c**) and storage modulus (**d**-**f**) versus angular frequency of the neat polymers (0 CNC) and nanocomposites (1 and 3 CNC) from solution casting. Filled and empty symbols are SAOS data before and after stress growth experiments (sh), respectively.

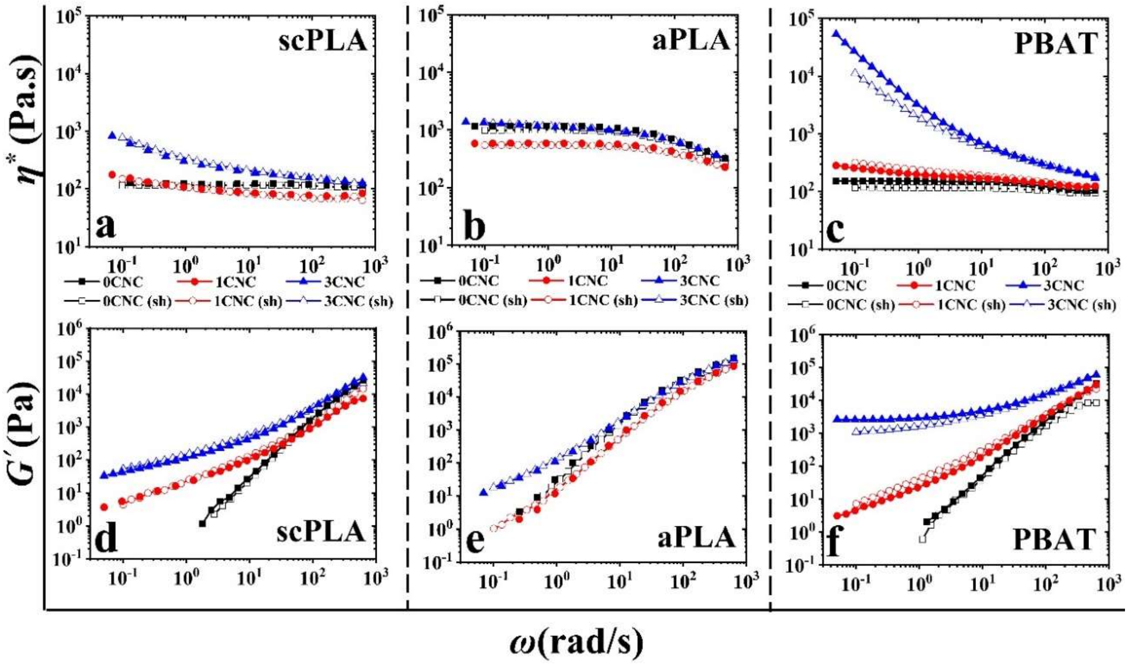

Figure S6. Effect of melt mixing (solvent casting+IMM) on the complex viscosity (**a**-**c**) and storage modulus (**d**-**f**) of the neat polymers (0 CNC) and nanocomposites (1 and 3 CNC) prepared through

solution casting as functions of angular frequency and CNC content. Filled and empty symbols are SAOS data before and after stress growth experiments (sh), respectively.

To confirm the absence of coalescence in the PLA/PBAT nanocomposites, time sweep experiments were conducted at a frequency of 1 rad/s for 1 h, and the results are presented in Figure S7. The initial increases of the complex viscosity could be due to the formation of an extended interconnected network of nanoparticles with time. If we look at the SAOS data (Figure 9 in the main manuscript), we observe a solid-like behavior at low frequencies when the CNCs are initially localized in the matrix or both phases, whereas this behavior is not observed when the CNCs are initially in PBAT. So, we can conclude that the proportion of CNCs at the interface between the matrix and droplet results in an interconnected network of nanoparticles over time. Due to PLA degradation and PBAT droplet coalescence, the time to reach a 10% drop in the complex viscosity is about 12 min and 60 min for scPLA/PBAT and aPLA/PBAT, respectively. In the presence of 1 wt% CNCs, when the CNCs were initially localized in the matrix or both phases, the system is stable up to 60 min. On the other hand, localizing CNCs in the dispersed phase (upward triangle) results in a decrease in the complex viscosity. However, this decrease is less than 10% for aPLA/(PBAT-1CNC) and around 10% for scPLA/(PBAT-1CNC) blend nanocomposites. For example, the viscosity of aPLA/(PBAT-1CNC) decreases from 3250 Pa.s to 3000 Pa.s, which is around 9% after 1 h.

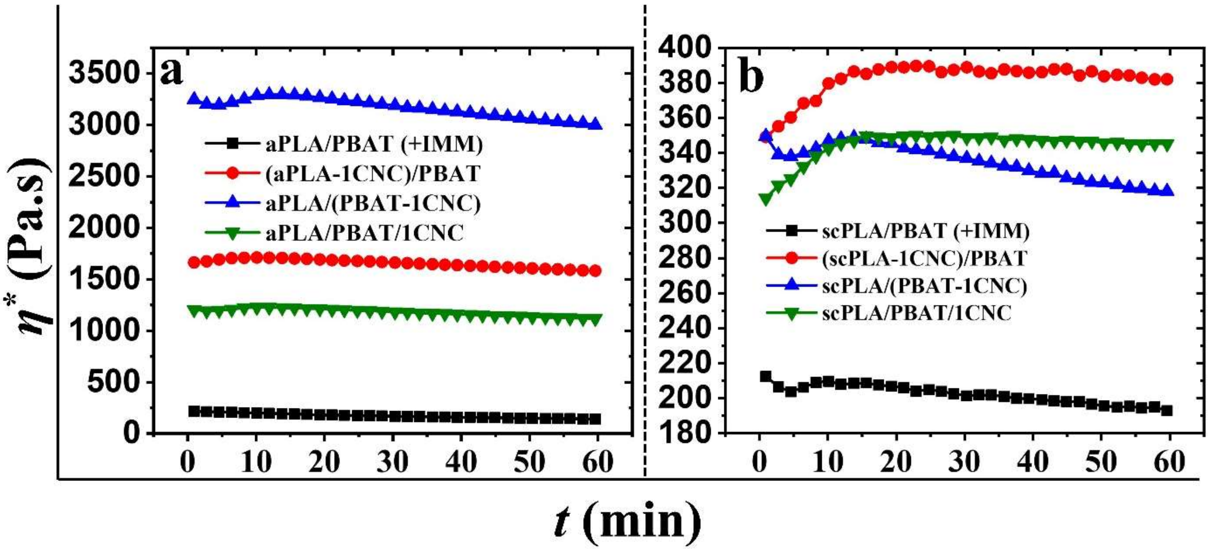

Figure S7. Complex viscosity (η^*) versus time (t) of the neat PLA/PBAT (**a**: amorphous and **b**: semicrystalline) and blend nanocomposites reinforced with 1 wt% CNCs during 1 h at a frequency of 1 rad/s and strain amplitude of 0.001.

References

1. Hansen, C. M. The Three Dimensional Solubility Parameter and Solvent Diffusion Coefficient. *J. Paint Technol.* **1967**, *39*, 104–117.
2. Bruel, C.; Beuguel, Q.; Tavares, J. R.; Carreau, P. J.; Heuzey, M.C. The Apparent Structural Hydrophobicity of Cellulose Nanocrystals. *J. Sci. Technol. For. Prod. Process.* **2018**, *7* (4), 13–23.
3. Bruel, C.; Tavares, J. R.; Carreau, P. J.; Heuzey, M. C. The Structural Amphiphilicity of Cellulose Nanocrystals Characterized from Their Cohesion Parameters. *Carbohydr. Polym.* **2019**, *205*, 184–191.
4. Bruel, C.; Davies, T. S.; Carreau, P. J.; Tavares, J. R.; Heuzey, M.C. Self-Assembly Behaviors of Colloidal Cellulose Nanocrystals: A Tale of Stabilization Mechanisms. *J. Colloid Interface Sci.* **2020**, *574*, 399–409.
5. Bruel, C.; Tavares, J. R.; Carreau, P. J.; Heuzey, M. Impact of Colloidal Stability on Cellulose Nanocrystals

Self-Ordering in Thin Films. *TechConnect Briefs* **2019**, No. 1, 61–64.

6. Abbott, S.; Hansen, C. M.; Yamamoto, H. *Hansen Solubility Parameters in Practice Complete with EBook , Software and Data*; 2013.
7. Mohammadi, M.; Bruel, C.; Heuzey, M. C.; Carreau, P. J. CNC Dispersion in PLA and PBAT Using Two Solvents: Morphological and Rheological Properties. *Cellulose* **2020**, *27* (17), 9877–9892.
8. HSP and Temperature | Hansen Solubility Parameters https://www.hansen-solubility.com/HSP-science/HSP-T.php (accessed Feb 23, 2021).
9. Bagheriasl, D.; Carreau, P. J.; Riedl, B.; Dubois, C.; Hamad, W. Y. Shear Rheology of Polylactide (PLA)–Cellulose Nanocrystal (CNC) Nanocomposites. *Cellulose* **2016**, *23* (3), 1885–1897.
10. Morelli, C. L.; Belgacem, M. N.; Branciforti, M. C.; C. B. Salon, M.; Bras, J.; Bretas, R. E. S. Nanocomposites of PBAT and Cellulose Nanocrystals Modified by in Situ Polymerization and Melt Extrusion. *Polym. Eng. Sci.* **2016**, *56* (12), 1339–1348.
11. Hansen, C. M. *Hansen Solubility Parameters*; CRC Press: Boca Raton, 2007.
12. HSPiP FAQ | Hansen Solubility Parameters https://www.hansen-solubility.com/HSPiP/faq.php (accessed Feb 23, 2021).
13. Fenouillot, F.; Cassagnau, P.; Majesté, J.-C. Uneven Distribution of Nanoparticles in Immiscible Fluids: Morphology Development in Polymer Blends. *Polymer (Guildf).* **2009**, *50* (6), 1333–1350.
14. Wu, S. *Polymer Interface and Adhesion*; Routledge, 2017.
15. Jalali Dil, E.; Favis, B. D. Localization of Micro- and Nano-Silica Particles in Heterophase Poly(Lactic Acid)/Poly(Butylene Adipate-Co-Terephthalate) Blends. *Polymer (Guildf).* **2015**, *76*, 295–306.
16. Jalali Dil, E.; Arjmand, M.; Otero Navas, I.; Sundararaj, U.; Favis, B. D. Interface Bridging of Multiwalled Carbon Nanotubes in Polylactic Acid/Poly(Butylene Adipate- Co -Terephthalate): Morphology, Rheology, and Electrical Conductivity. *Macromolecules* **2020**, *53* (22), 10267–10277.
17. Khoshkava, V.; Kamal, M. R. Effect of Surface Energy on Dispersion and Mechanical Properties of Polymer/Nanocrystalline Cellulose Nanocomposites. *Biomacromolecules* **2013**, *14* (9), 3155–3163.
18. Wu, D.; Yuan, L.; Laredo, E.; Zhang, M.; Zhou, W. Interfacial Properties, Viscoelasticity, and Thermal Behaviors of Poly(Butylene Succinate)/Polylactide Blend. *Ind. Eng. Chem. Res.* **2012**, *51* (5), 2290–2298.
19. Palierne, J. F. Linear Rheology of Viscoelastic Emulsions with Interfacial Tension. *Rheol. Acta* **1990**, *29* (3), 204–214.
20. Wu, S. *Polymer Interface and Adhesion*; Routledge: New York ;;Basel, 2017.
21. Bousmina, M.; Muller, R. Linear Viscoelasticity in the Melt of Impact PMMA. Influence of Concentration and Aggregation of Dispersed Rubber Particles. *J. Rheol. (N. Y. N. Y).* **1993**, *37* (4), 663–679.
22. Lacroix, C.; Bousmina, M.; Carreau, P. J.; Favis, B. D.; Michel, A. Properties of PETG/EVA Blends: 1. Viscoelastic, Morphological and Interfacial Properties. *Polymer (Guildf).* **1996**, *37* (14), 2939–2947.
